# Supplementary material for: Yeti claws: Cheliped sexual dimorphism and symmetry in deep-sea yeti crabs (Kiwaidae)
Source: PLoS One. 2025 Feb 5;20(2):e0314320. doi: 10.1371/journal.pone.0314320 (PMC11798501; doi:10.1371/journal.pone.0314320)
Supplement: S1 Table — CL = carapace length. PL = cheliped propodus length. PH = cheliped propodus height. M = male. F = female. (DOCX) [file pone.0314320.s001.docx]

**Table S1.** Measurements (mm) for *Kiwa tyleri* body parts in this study to nearest 0.1 mm. CL= carapace length. PL = cheliped propodus length. PH = cheliped propodus height. M = male. F = female.

| **Sex** | **CL** | **PL Right** | **PH Right** | **PL Left** | **PH Left** |
| --- | --- | --- | --- | --- | --- |
| M | 45.5 | 23.2 | 15.1 | 23 | 15.1 |
| M | 66 | 40.4 | 24.1 | 41.1 | 23.9 |
| M | 68.6 | 42.2 | 24.6 | 42.3 | 24.7 |
| M | 19.8 | 9.4 | 5.6 | 9.4 | 5.7 |
| M | 20.9 | 9.1 | 6 |  |  |
| M | 13.7 | 6.4 | 3.9 |  |  |
| F | 21.9 | 9.5 | 5.6 | 9.5 | 5.3 |
| F | 20.4 | 8.8 | 5.2 | 8.8 | 5.4 |
| F | 24.5 | 10.5 | 5.9 | 10.5 | 5.6 |
| F | 54 | 27.5 | 15.5 | 28.7 | 15.7 |
| M | 27.1 | 12.2 | 8.4 | 12.4 | 8.4 |
| M | 23.4 | 10.3 | 7.4 | 10.8 | 7.4 |
| M | 21.5 | 10 | 6.6 | 9.7 | 6.5 |
| M | 21 | 8.4 | 5.3 | 8.8 | 4.9 |
| M | 22.2 | 10.3 | 6.6 | 10.7 | 6.6 |
| M | 68.5 | 44.5 | 27.1 | 44.6 | 26.5 |
| F | 38.4 | 17.5 | 10.7 | 18.4 | 10.7 |
| M | 46.2 | 23.2 | 14.5 | 23.4 | 13.2 |
| F | 52.7 | 26.2 | 14.7 | 26.9 | 14.2 |
| M | 53.5 | 34.8 | 19.5 | 34.9 | 19.7 |
| M | 51.4 | 29.2 | 17.4 | 28.8 | 17 |
| M | 58.2 | 34.9 | 20.2 | 35.5 | 20.4 |
| M | 37.6 | 19.8 | 12 | 19.9 | 12.2 |
| M | 54.8 | 31 | 18.8 | 30.7 | 18.9 |
| M | 35 | 18.6 | 10.6 | 18.6 | 10.6 |
| F | 34.9 | 15 | 10.2 | 17.1 | 9.9 |
| F | 32.2 | 14.1 | 8.4 | 15.3 | 9.2 |
| F | 37 | 18.2 | 10.1 | 18.2 | 10.1 |
| F | 30.9 | 15 | 8.4 | 15.2 | 8.4 |
| F | 35.3 | 16.8 | 9.2 | 17.8 | 10.2 |
| F | 39.2 | 18.9 | 10 | 18.9 | 11.1 |
| F | 38.5 | 17.3 | 11 | 17.9 | 11 |
| F | 41.6 | 19.5 | 11.8 | 19.9 | 11.8 |
| F | 34.6 | 16.2 | 8.9 | 16.4 | 9.1 |
| F | 35.5 | 17.9 | 10.4 | 17.7 | 10.5 |
| F | 28.9 | 14.1 | 8 | 14.1 | 7.9 |
| F | 41.6 | 20.5 | 12.4 | 20.5 | 12 |
| F | 35 | 16.9 | 9.5 | 16.2 | 9.8 |
| F | 38.2 | 18.5 | 10.4 | 18.5 | 10.6 |
| F | 35.9 | 17 | 9.8 | 16.2 | 9.4 |
| F | 19.7 | 9.3 | 5.4 | 9.7 | 5.4 |
| F | 37.8 | 18.3 | 10.3 | 17.6 | 10.1 |
| F | 43.5 | 22.1 | 12.7 | 21.4 | 12.8 |
| F | 41.4 | 19.2 | 12.4 | 19.5 | 12.5 |
| F | 38.1 | 18.5 | 10.4 | 18.4 | 11.2 |
| M | 41.6 | 24.7 | 14.9 | 25 | 15.1 |
| M | 35.2 | 18.6 | 11.1 | 18.7 | 11.2 |
| F | 40.2 | 19.4 | 11.5 | 20.1 | 11.6 |
| F | 20.8 | 10.1 | 4.5 | 10.2 | 5.4 |
| M | 72.5 | 49.6 | 26.9 | 50.5 | 27.8 |
| M | 67 | 48.8 | 27.6 | 48.7 | 26.5 |
| M | 37.5 | 19.7 | 13.4 | 19.3 | 13.3 |
| M | 35.8 | 17.6 | 11.7 | 17.5 | 11.5 |
| M | 40.4 | 23.2 | 13.6 | 23.2 | 14.1 |
| M | 33.2 | 18.7 | 10.9 | 18.6 | 10.9 |
| M | 46.7 | 26.4 | 15.7 | 26.5 | 15.4 |
| M | 47.7 | 26.3 | 17.6 | 26.5 | 17.2 |
| M | 53.7 | 31.1 | 18.7 | 31.4 | 18.5 |
| M | 55 | 31.6 | 18.7 | 31.7 | 18.6 |
| M | 71 |  |  | 51.5 | 29.4 |
| F | 21.1 | 8.6 | 5.5 | 8.7 | 5.1 |
| F | 17.3 | 8 | 4.4 | 8 | 4.7 |
| F | 22.4 | 10 | 5.7 | 9.6 | 5.7 |
| F | 18.1 | 8.2 | 4.6 | 8.1 | 4.7 |
| F | 21.1 | 9.6 | 5.1 |  |  |
| F | 22.7 |  |  | 10.5 | 6.4 |
| F | 25.3 |  |  | 9.5 | 5.4 |
| F | 19.8 |  |  | 9.1 | 4.9 |
| F | 21.2 | 10 | 5.2 | 9.8 | 5.3 |
| F | 17.5 | 8.2 | 4.5 | 8.1 | 4.6 |
| M | 38.2 | 23.2 | 12.8 | 23.2 | 12.6 |
| M | 45.5 | 28.7 | 16.3 | 28.7 | 16.1 |
| M | 24.8 |  |  | 11.7 | 7 |
| M | 24.1 |  |  | 11.1 | 7.5 |
| M | 16.2 |  |  | 7.2 | 4.6 |
| M | 45.3 | 24.8 | 16 | 24.8 | 16.2 |
| M | 19.8 | 9.2 | 5.4 | 9.5 | 5.8 |
| M | 21.4 | 10.6 | 6.6 | 10.5 |  |
| M | 18.6 |  |  | 8.5 | 5.2 |
| F | 37 | 17.9 | 10.2 | 18.4 | 10.1 |
| F | 33.2 | 15 | 9.3 | 15.5 | 9 |
| F | 18 | 8.2 | 4.6 | 8.1 | 4.6 |
| F | 20.7 | 9.2 | 5.9 | 9.5 | 5.9 |
| F | 17 | 7 | 4 |  |  |
| F | 19.9 |  |  | 9.1 | 5.2 |
| F | 15.2 | 7.1 | 3.9 | 7.2 | 3.9 |
| F | 18.8 | 8.8 | 5 | 8.4 | 4.5 |
| F | 38.6 |  |  | 18.4 | 10.9 |
| F | 35.6 | 18.2 | 10.4 | 17.6 | 10 |
| M | 47.3 |  |  | 29.2 | 16.4 |
| M | 31.8 |  |  | 14.7 | 8.8 |
| M | 30.4 | 15.6 | 10.5 | 15.7 | 10.4 |
| M | 40.9 | 23.8 | 14.8 | 23.6 | 14.7 |
| F | 22.9 | 9 | 5.5 | 10.4 | 6.1 |
| F | 23.5 |  |  | 9.6 | 5.7 |
| F | 24.9 |  |  | 11 | 6.3 |
| F | 26.1 | 11.6 | 6.7 |  |  |
| F | 18.4 | 8.2 | 4.6 | 8.7 | 4.7 |
| F | 24.5 | 9.4 | 4.6 |  |  |
| F | 24 | 11.2 | 6.2 | 10.7 | 5.8 |
| F | 21.4 |  |  | 9.2 | 5.6 |
| F | 25.7 | 12.2 | 5.6 |  |  |
| F | 24.7 |  |  | 11.6 | 6.7 |
| F | 22.9 | 9.5 | 6.1 | 9.6 | 6.1 |
| F | 20.1 | 9.6 | 5.7 |  |  |
| F | 21.8 | 9.9 | 6.1 | 9.9 | 6.1 |
| F | 26.9 | 11.4 | 7.4 | 11.3 | 6.9 |
| M | 33.5 | 16.2 | 11.7 | 16.1 | 11 |
| M | 26.6 | 13 | 8.2 | 12.9 | 8.2 |
| M | 38.7 | 19.7 | 13 | 19.3 | 13.2 |
| M | 35.6 | 19.4 | 11.5 | 18.8 | 11.7 |
| M | 39.9 | 21.4 | 13.8 | 22.2 | 13.2 |
| M | 52 | 31.2 | 18.1 | 31.3 | 17.8 |
| M | 32.4 | 16.6 | 11.2 | 16.3 | 11.2 |
| M | 48.2 | 29.6 | 16.4 |  |  |
| M | 42.6 | 23.9 | 15.2 | 23.7 | 15.2 |
| M | 38.8 | 20.8 | 14.2 | 21 | 14 |
| M | 40.4 | 21.6 | 14.3 | 21.2 | 14.2 |
| M | 33.8 | 16.6 | 11 | 16.3 | 10.9 |
| M | 30.5 | 16.1 | 9.7 | 16.2 | 9.3 |
| M | 47.7 | 27.4 | 16.4 | 27.5 | 15.5 |
| M | 31.4 |  |  | 14.5 | 10.3 |
| M | 41.9 | 22.7 | 14.5 | 22.8 | 14.2 |
| M | 22.7 | 10.7 | 7.2 | 10.8 | 7.4 |
| M | 16.6 | 7.7 | 4.3 | 7.7 | 4.2 |
| F | 31.3 | 14.8 | 8.8 | 14.8 | 8.7 |
| F | 38.3 | 18 | 10.6 | 18.7 | 10.3 |
| F | 42.5 | 14 | 8.6 | 20.2 | 11.7 |
| F | 39.5 | 18.4 | 12.4 | 18.6 | 11.8 |
| F | 38.7 | 20.4 | 11.6 | 20.4 | 11.3 |
| M | 39.3 | 20.8 | 14 | 20.9 | 13.6 |
| M | 37.4 | 20.2 | 12.3 | 20.4 | 12.5 |
| M | 35.4 | 17.3 | 11.5 | 16.9 | 10.5 |
| M | 35 | 18 | 11.7 | 18.3 | 11.6 |
| M | 30.9 | 13.9 | 8.1 | 16.8 | 9.6 |
