# Supplementary material for: Yeti claws: Cheliped sexual dimorphism and symmetry in deep-sea yeti crabs (Kiwaidae)
Source: PLoS One. 2025 Feb 5;20(2):e0314320. doi: 10.1371/journal.pone.0314320 (PMC11798501; doi:10.1371/journal.pone.0314320)
Supplement: S2 Table — Models initially compared slope angle between males and females and if non-significant, a comparison with slope elevation (y-axis offset) assuming equal slopes was made. 95% CI = 95% confidence interval, R2 = regression model coefficient of determination, P1 = p-value for regression models against null hypothesis that variables are uncorrelated, Null = null hypothesis of test comparing male and female slopes angle/elevation, P2 = p-value, indicating significance of test with values < 0.05 highlighted in bold. (DOCX) [file pone.0314320.s002.docx]

**Table S2.** Standardised major axis (SMA) regression model output showing the relationship between log-transformed propodus length/height and carapace length for male and female *Kiwa tyleri* and *Kiwa puravida* specimens as performed by the smatr R package. Models initially compared slope angle between males (M) and females (F) and if non-significant, a comparison with slope elevation (y-axis offset) assuming equal slopes was made. 95% CI = 95% confidence interval, R^2^ = regression model coefficient of determination, P1 = p-value for regression models against null hypothesis that variables are uncorrelated, Null = null hypothesis of test comparing male and female slopes angle/elevation, P2 = p-value indicating significance of test with values < 0.05 highlighted in bold.

| **Species** | **Model type** | **Comparison** | **Estimate** | **95% CI** | **R^2^** | **P1** | **Null** | **Statistic** | **P2** |
| --- | --- | --- | --- | --- | --- | --- | --- | --- | --- |
| ***Kiwa tyleri*** | log propodus length ~ log carapace length * sex | slope (F) | 1.130 | 1.088 – 1.175 | 0.976 | 0.000 | equal slopes | likelihood ratio 24.03 (1df) | **0.000** |
|  |  | slope (M) | 1.284 | 1.245 – 1.323 | 0.985 | 0.000 |  |  |  |
|  |  | intercept (F) | -0.520 | -0.583 – -0.456 | 0.976 | 0.000 |  |  |  |
|  |  | intercept (M) | -0.713 | -0.774 – -0.652 | 0.985 | 0.000 |  |  |  |
|  | log propodus height ~ log carapace length * sex | slope (F) | 1.165 | 1.112 – 1.222 | 0.964 | 0.000 | equal slopes | likelihood ratio 3.1 (1df) | 0.078 |
|  |  | slope (M) | 1.224 | 1.191 – 1.257 | 0.988 | 0.000 |  |  |  |
|  |  | intercept (F) | -0.810 | -0.890 – -0.730 | 0.964 | 0.000 |  |  |  |
|  |  | intercept (M) | -0.828 | -0.880 – -0.776 | 0.988 | 0.000 |  |  |  |
|  | log propodus height ~ log carapace length + sex | slope (F) | 1.209 | 1.180 – 1.238 | 0.964 | 0.000 | equal elevation | wald statistic 174.9 (1df) | **0.000** |
|  |  | slope (M) | 1.209 | 1.180 – 1.238 | 0.988 | 0.000 |  |  |  |
|  |  | intercept (F) | -0.873 | -0.915 – -0.831 | 0.964 | 0.000 |  |  |  |
|  |  | intercept (M) | -0.806 | -0.851 – -0.761 | 0.988 | 0.000 |  |  |  |
| ***Kiwa puravida*** | log propodus length ~ log carapace length * sex | slope (F) | 1.088 | 1.059 – 1.118 | 0.988 | 0.000 | equal slopes | likelihood ratio 0.004 (1df) | 0.951 |
|  |  | slope (M) | 1.087 | 1.063 – 1.112 | 0.982 | 0.000 |  |  |  |
|  |  | intercept (F) | -0.287 | -0.313 – -0.262 | 0.988 | 0.000 |  |  |  |
|  |  | intercept (M) | -0.275 | -0.293 – -0.256 | 0.982 | 0.000 |  |  |  |
|  | log propodus length ~ log carapace length + sex | slope (F) | 1.087 | 1.069 – 1.106 | 0.988 | 0.000 | equal elevation | wald statistic 1.179 (1df) | 0.277 |
|  |  | slope (M) | 1.087 | 1.069 – 1.106 | 0.982 | 0.000 |  |  |  |
|  |  | intercept (F) | -0.287 | -0.308 – -0.266 | 0.988 | 0.000 |  |  |  |
|  |  | intercept (M) | -0.275 | -0.292 – -0.258 | 0.982 | 0.000 |  |  |  |
|  | log propodus height ~ log carapace length * sex | slope (F) | 1.074 | 1.041 – 1.107 | 0.984 | 0.000 | equal slopes | likelihood ratio 0.668 (df) | 0.414 |
|  |  | slope (M) | 1.091 | 1.067 – 1.115 | 0.983 | 0.000 |  |  |  |
|  |  | intercept (F) | -0.720 | -0.748 – -0.691 | 0.984 | 0.000 |  |  |  |
|  |  | intercept (M) | -0.737 | -0.756 – -0.719 | 0.983 | 0.000 |  |  |  |
|  | log propodus height ~ log carapace length + sex | slope (F) | 1.085 | 1.065 – 1.104 | 0.984 | 0.000 | equal elevation | wald statistic 0.381 (1df) | 0.537 |
|  |  | slope (M) | 1.085 | 1.065 – 1.104 | 0.983 | 0.000 |  |  |  |
|  |  | intercept (F) | -0.727 | -0.750 – -0.704 | 0.984 | 0.000 |  |  |  |
|  |  | intercept (M) | -0.734 | -0.751 – -0.717 | 0.983 | 0.000 |  |  |  |
