# Supplementary material for: Yeti claws: Cheliped sexual dimorphism and symmetry in deep-sea yeti crabs (Kiwaidae)
Source: PLoS One. 2025 Feb 5;20(2):e0314320. doi: 10.1371/journal.pone.0314320 (PMC11798501; doi:10.1371/journal.pone.0314320)
Supplement: Attachment — Submitted filename: PONE-D-24-41657_reviewer.pdf [file pone.0314320.s003.pdf]

PLOS ONE

Fighting for Favour: Sexual Dimorphism and Symmetry in the Hydrothermal Vent and Methane Seep Endemic Yeti Crabs (Kiwaidae).

--Manuscript Draft--

**Manuscript Number:**	PONE-D-24-41657
**Article Type:**	Research Article
**Full Title:**	Fighting for Favour: Sexual Dimorphism and Symmetry in the Hydrothermal Vent and Methane Seep Endemic Yeti Crabs (Kiwaidae).
**Short Title:**	Fighting for Favour: Sexual Dimorphism and Symmetry in the Yeti Crabs (Kiwaidae).
**Corresponding Author:**	Christopher Nicolai Roterman, DPhil<br>University of Portsmouth<br>Portsmouth, Hampshire UNITED KINGDOM OF GREAT BRITAIN AND NORTHERN IRELAND
**Keywords:**	Deep sea; yeti crab; hoff crab; kiwaidae; anomura; chirostyloidea; squat lobster; crustacean; decapod; evolution; sexual selection; sexual dimorphism; cheliped; claw; morphology; competition, behaviour; hydrothermal vent; methane seep; hydrocarbon seep; east scotia ridge; Costa Rica; south sandwich islands; Atlantic; southern ocean; pacific; chemosynthesis; chemosymbiosis; ectosymbiosis; chemosynthetic; black smokers
**Abstract:**	Yeti crabs (Kiwaidae) are deep-sea hydrothermal vent and methane seep dwelling crustaceans that farm chemosynthetic microbes on their bodies. Sexual dimorphism is a common feature of decapod crustaceans, but little is known about its prevalence in species from deep-sea habitats. We address this knowledge deficit by investigating sexual dimorphism and body symmetry in the hydrothermal-vent endemic 'Hoff crab', Kiwa tyleri, focusing on claw (cheliped) morphology. A total of 135 specimens from the East Scotia Ridge were examined, revealing a high degree of cheliped propodus symmetry, consistent with previous findings suggesting their claws are primarily used for microbial farming and intraspecific competition, but not predation. Both male and female chelipeds exhibit positive allometry with increasing carapace length, but males possess significantly larger chelipeds compared with females when accounting for carapace size, exhibiting faster growing cheliped carpus and propodus length, and broader carpus and propodus heights throughout the size distribution. This marked difference is the strongest evidence of sexually selected dimorphism in Kiwaidae, with males likely using their larger chelipeds in competitive interactions with other males for mating access to females, as observed in other decapod crustaceans. In contrast, a reanalysis of data for the methane seep inhabiting yeti crab Kiwa puravidarevealed no significant sexual dimorphism in cheliped proportions when accounting for body size, indicating a possible lack of similar sexual selection pressures, and highlighting potential key differences in the ecological and reproductive strategies of Kiwa tyleri and Kiwa puravida relating to cheliped function, microbial productivity and population density. This research enhances our understanding of the role of sexual selection and morphological specialization in yeti crabs, providing a basis for future studies of their evolutionary biology and ecology.
**Order of Authors:**	Christopher Nicolai Roterman, DPhil
	Molly McArthur, MSc
	Cecilia Laverty Baralle, BSc (Hons)
	Leigh Marsh, PhD
	Jon T Copley, PhD
**Additional Information:**	
**Question**	**Response**
**Financial Disclosure**	Yes

Powered by Editorial Manager® and ProduXion Manager® from Aries Systems Corporation

Enter a financial disclosure statement that describes the sources of funding for the work included in this submission. Review the submission guidelines for detailed requirements. View published research articles from *PLOS ONE* for specific examples.<br><br>This statement is required for submission and **will appear in the published article** if the submission is accepted. Please make sure it is accurate.<br><br>**Funded studies**<br>Enter a statement with the following details:<br>• Initials of the authors who received each award<br>• Grant numbers awarded to each author<br>• The full name of each funder<br>• URL of each funder website<br>• Did the sponsors or funders play any role in the study design, data collection and analysis, decision to publish, or preparation of the manuscript?<br><br>Did you receive funding for this work?	
Please add funding details.<br>as follow-up to "**Financial Disclosure**<br><br>Enter a financial disclosure statement that describes the sources of funding for the work included in this submission. Review the submission guidelines for detailed requirements. View published research articles from *PLOS ONE* for specific examples.<br><br>This statement is required for submission and **will appear in the published article** if the submission is accepted. Please make sure it is accurate.	Specimens were collected with a NERC (UK) Consortium Grant (NE/D01249X/1; ChEsSo)

Powered by Editorial Manager® and ProduXion Manager® from Aries Systems Corporation

**Funded studies**<br>Enter a statement with the following details:<br>• Initials of the authors who received each award<br>• Grant numbers awarded to each author<br>• The full name of each funder<br>• URL of each funder website<br>• Did the sponsors or funders play any role in the study design, data collection and analysis, decision to publish, or preparation of the manuscript?<br><br>Did you receive funding for this work?"	
Please select the country of your main research funder (please select carefully as in some cases this is used in fee calculation).<br>as follow-up to "**Financial Disclosure**<br><br>Enter a financial disclosure statement that describes the sources of funding for the work included in this submission. Review the submission guidelines for detailed requirements. View published research articles from *PLOS ONE* for specific examples.<br><br>This statement is required for submission and **will appear in the published article** if the submission is accepted. Please make sure it is accurate.<br><br>**Funded studies**<br>Enter a statement with the following details:<br>• Initials of the authors who received each award<br>• Grant numbers awarded to each author<br>• The full name of each funder<br>• URL of each funder website<br>• Did the sponsors or funders play any role in the study design, data collection and analysis, decision to publish, or preparation of the manuscript?<br><br>Did you receive funding for this work?"	UNITED KINGDOM - GB
**Competing Interests**	The authors have declared that no competing interests exist.

Use the instructions below to enter a competing interest statement for this submission. On behalf of all authors, disclose any competing interests that could be perceived to bias this work—acknowledging all financial support and any other relevant financial or non-financial competing interests.<br><br>This statement is required for submission and **will appear in the published article** if the submission is accepted. Please make sure it is accurate and that any funding sources listed in your Funding Information later in the submission form are also declared in your Financial Disclosure statement.<br><br>View published research articles from *PLOS ONE* for specific examples.<br><br>**NO authors have competing interests**<br><br>Enter: *The authors have declared that no competing interests exist.*<br><br>**Authors with competing interests**<br><br>Enter competing interest details beginning with this statement:<br><br>*I have read the journal's policy and the authors of this manuscript have the following competing interests: [insert competing interests here]*<br><br>* typeset	
**Ethics Statement**<br><br>Enter an ethics statement for this submission. This statement is required if the study involved:<br><br>• Human participants<br>• Human specimens or tissue<br>• Vertebrate animals or cephalopods<br>• Vertebrate embryos or tissues	This srtudy involved the sampling of deep-sea organisms on the NERC-funded research cruise JC042 aboard the RRS James Cook, with the University of Southampton.

Powered by Editorial Manager® and ProduXion Manager® from Aries Systems Corporation

• Field research<br><br>Write "N/A" if the submission does not require an ethics statement.<br><br>General guidance is provided below. Consult the submission guidelines for detailed instructions. **Make sure that all information entered here is included in the Methods section of the manuscript.**	

Powered by Editorial Manager® and ProduXion Manager® from Aries Systems Corporation

**Format for specific study types**<br><br>**Human Subject Research (involving human participants and/or tissue)**<br>• Give the name of the institutional review board or ethics committee that approved the study<br>• Include the approval number and/or a statement indicating approval of this research<br>• Indicate the form of consent obtained (written/oral) or the reason that consent was not obtained (e.g. the data were analyzed anonymously)<br><br>**Animal Research (involving vertebrate animals, embryos or tissues)**<br>• Provide the name of the Institutional Animal Care and Use Committee (IACUC) or other relevant ethics board that reviewed the study protocol, and indicate whether they approved this research or granted a formal waiver of ethical approval<br>• Include an approval number if one was obtained<br>• If the study involved *non-human primates*, add *additional details* about animal welfare and steps taken to ameliorate suffering<br>• If anesthesia, euthanasia, or any kind of animal sacrifice is part of the study, include briefly which substances and/or methods were applied<br><br>**Field Research**<br><br>Include the following details if this study involves the collection of plant, animal, or other materials from a natural setting:<br>• Field permit number<br>• Name of the institution or relevant body that granted permission	
**Data Availability**<br><br>Authors are required to make all data underlying the findings described fully available, without restriction, and from the time of publication. PLOS allows rare exceptions to address legal and ethical concerns. See the PLOS Data Policy and FAQ for detailed information.	Yes - all data are fully available without restriction

A Data Availability Statement describing where the data can be found is required at submission. Your answers to this question constitute the Data Availability Statement and **will be published in the article**, if accepted.<br><br>**Important:** Stating 'data available on request from the author' is not sufficient. If your data are only available upon request, select 'No' for the first question and explain your exceptional situation in the text box.<br><br>Do the authors confirm that all data underlying the findings described in their manuscript are fully available without restriction?	
**Describe where the data may be found in full sentences. If you are copying our sample text, replace any instances of XXX with the appropriate details.**<br><br>• If the data are **held or will be held in a public repository**, include URLs, accession numbers or DOIs. If this information will only be available after acceptance, indicate this by ticking the box below. For example: *All XXX files are available from the XXX database (accession number(s) XXX, XXX.).*<br>• If the data are all contained **within the manuscript and/or Supporting Information files**, enter the following: *All relevant data are within the manuscript and its Supporting Information files.*<br>• If neither of these applies but you are able to provide **details of access elsewhere**, with or without limitations, please do so. For example:<br><br>*Data cannot be shared publicly because of [XXX]. Data are available from the XXX Institutional Data Access / Ethics Committee (contact via XXX) for researchers who meet the criteria for access to confidential data.*<br><br>*The data underlying the results presented in the study are available from (include the name of the third party*	Raw data are included in the supporting information.

and contact information or URL).<br>• This text is appropriate if the data are owned by a third party and authors do not have permission to share the data.<br><br>* typeset	
Additional data availability information:	

Manuscript

Click here to access/download;Manuscript;Kiwa_dimorphism_submission_1

Fighting for Favour: Sexual Dimorphism and Symmetry in the Hydrothermal Vent and Methane Seep Endemic Yeti Crabs (Kiwaidae).

Christopher Nicolai Roterman[1, *], Molly McArthur[1], Cecilia Laverty Baralle[1], Leigh Marsh[2], Jon T Copley[2]

1 Institute of Marine Science, University of Portsmouth, Portsmouth, PO4 9LY, UK

2 Ocean and Earth Science, University of Southampton, Waterfront Campus, Southampton SO14 3ZH, UK,

[*]Corresponding author

Email: nicolai.roterman@port.ac.uk

Abstract

Yeti crabs (Kiwaidae) are deep-sea hydrothermal vent and methane seep dwelling crustaceans that farm chemosynthetic microbes on their bodies. Sexual dimorphism is a common feature of decapod crustaceans, but little is known about its prevalence in species from deep-sea habitats. We address this knowledge deficit by investigating sexual dimorphism and body symmetry in the hydrothermal-vent endemic 'Hoff crab', *Kiwa tyleri*, focusing on claw (cheliped) morphology. A total of 135 specimens from the East Scotia Ridge were examined, revealing a high degree of cheliped propodus symmetry, consistent with previous findings suggesting their claws are primarily used for microbial farming and intraspecific competition, but not predation. Both male and female chelipeds exhibit positive allometry with increasing carapace length, but males possess significantly larger chelipeds compared with females when accounting for carapace size, exhibiting faster growing cheliped carpus and propodus length, and broader carpus and propodus heights throughout the size distribution. This marked difference is the strongest evidence of sexually selected dimorphism in Kiwaidae, with males likely using their larger chelipeds in competitive interactions with other males for mating access to females, as observed in other decapod crustaceans. In contrast, a reanalysis of data for the methane seep inhabiting yeti crab *Kiwa puravida* revealed no significant sexual dimorphism in cheliped proportions when accounting for body size, indicating a possible lack of similar sexual selection pressures, and highlighting potential key differences in the ecological and reproductive strategies of *Kiwa tyleri* and *Kiwa puravida* relating to cheliped function, microbial productivity and population density. This research enhances our understanding of the role of sexual selection and morphological specialization in yeti crabs, providing a basis for future studies of their evolutionary biology and ecology.

Introduction

Deep-sea Chemosynthetic Ecosystems

In contrast to the general pattern of photosynthetically derived food limitation in the deep sea, hydrothermal vents and hydrocarbon (methane) seeps host high biomass oases of endemic macrofauna sustained by the primary productivity of chemosynthetic microbes (1). These communities, first discovered in 1977 (2) experience steep environmental gradients in the mixing zone between ambient seawater and fluids emanating from the seafloor, which are typically anoxic, acidic, and in the case of hydrothermal vents very hot (temperatures > 350°C) and rich in dissolved metals and other compounds (1). Consequently, macrofauna recovered from these habitats exhibit a variety of physiological adaptations to tolerate hypoxia (3,4), thermal stress, protection and detection (5–12), and toxicity (13,14). Strikingly, these animals have often developed intimate symbioses with chemosynthetic microbes (15), either farming microbes on modified appendages (16–18) or internally (19–22), ensuring a reliable and plentiful food source. However, owing to the remoteness of such habitats, the challenges inherent in their sampling and long-term monitoring, and difficulties in maintaining specimens in aquaria, little is known about the behaviour, ecology and life-history of these animals compared with shallow-water species.

Yeti Crabs

One such example are the yeti crabs (Kiwaidae): a decapod crustacean family within the infraorder Anomura, first discovered in 2005 (23) and found exclusively at deep-sea chemosynthetic ecosystems, comprising five described species within the genus *Kiwa* (23–27) and one undescribed (28) from the Pacific, Southern and Indian Oceans. These chirostyloid squat lobsters likely radiated within the last 45 million years (28,29) and appear to be sustained primarily by farming chemosynthetic microbes on hair-like setae extensively covering their

exoskeleton (hence their common name), as indicated by the morphological examination of setae (27,30,31) feeding appendages (24–27), and isotope analyses (32–34). All species appear to lack eyes, (23–27) and the basal bauplan for the family is individuals exhibiting disproportionately long, slender chelipeds with plumose or bristly setae (28) (Figure 1). An exception to this is *Kiwa tyleri* from the East Scotia Ridge in the Southern Ocean, commonly known as the 'Hoff crab', and also a closely-related undescribed species *Kiwa* sp. SWIR, which have a more compact overall shape, shorter chelipeds and a greater profusion of setae on their ventral surface than on the chelipeds (28). *Kiwa tyleri* was observed in exceptionally high densities (> 1000 individuals m^{-2}) at the base of vent chimneys (Figure 2), with larger individuals up the sides of the chimneys (35,36), living within a thermal envelope bounded by ambient sea temperatures ranging from 0˚C to -1.3˚C (35), which are typically too cold for crab and lobster survival (37–39). It has been hypothesised, therefore, that the distinctive body shape and placement of setae in this species is an adaptation to limited space and the vertical terrain on which *Kiwa tyleri* must survive, constrained within a thermal bubble (25,40). Yeti crabs produce relatively few but large lecithotrophic larvae, exhibiting extremely abbreviated development and hatching as megalopa following a likely prolonged brooding period (40,41). Their mode of dispersal is thereby less optimised for long-range dispersal than local retention on patchy habitats, which may partly explain the extreme isolation of some species (28).

Figure 1. Dorsal view of the known yeti crab (Kiwaidae) species, from Roterman et al. (28). Scale bars are approximate and represent 10 mm.

Figure 2. High density *Kiwa tyleri* assemblage in shimmering water hydrothermal vents at E9 on the East Scotia Ridge. From Rogers et al. (35).

At present, the longevity of kiwaid individuals, the age when they become reproductively active and their lifelong fecundity are unknown, but some aspects of reproductive behaviour have been inferred from spatial analyses. Spatial mapping and sampling of *Kiwa tyleri* at vents on the East

Scotia Ridge in the Southern Ocean indicate the largest individuals, which are found closest to the hot vent effluent, are males, with smaller, brooding females found more in the vent periphery, possibly trading access to microbial food production for more oxygenated conditions in which to brood their eggs (40,42). While this spatial pattern has only been observed in *Kiwa tyleri*, an extensive morphological study (> 200 specimens) of the tropical East Pacific methane seep-associated *Kiwa puravida* by Azofeifa-Solano et al. (43) found the largest individuals to be males, as did the recent species description of *Kiwa gemma* from vents on the Galapagos microplate (27), possibly indicating a similar spatial pattern occurs with those species. This apparent sexual dimorphism may be suggestive of sexual selection (44) or at least differential growth as a consequence of the environmental constraints that males and females experience as a function of their reproductive ecology (40).

Chelipeds & Sexual Dimorphism

Chelipeds are the first pair of walking appendages (pereopods) in decapod crustaceans with the final two segments comprising the claw (chela) with a movable finger (dactylus) and a larger fixed segment (propodus). These have a variety of uses, including as weapons of offense and defence, for predation, collecting suspended food particles, intraspecific competition for mates, visual display and generating sound (45). A common, but not exclusive feature amongst decapods is heterochely, where one claw is larger or differently shaped to the other and is related to task specialisation, such as having a crushing and a cutting claw (46,47), but may also in some species be sexually dimorphic and sexually selected (45,48). Within Kiwaidae, the chelipeds appear to be primarily used for farming microbes, based on their dense covering of setae, with the possible exception of *Kiwa tyleri* and *Kiwa* sp. SWIR as already mentioned. Thurber et al. (24) reported *Kiwa puravida* actively waving their chelipeds, presumably to enhance microbial production, although this behaviour has not been observed in the hydrothermal vent species. The chelae in *Kiwa puravida*, *Kiwa araonae* and *Kiwa gemma* which belong to the 'bristly' clade (28) are similar

in being slender, with a prominent tooth on the cutting edge of the dactylus and row of teeth on the cutting edge of the propodus, suggesting microbial production is not the only function. Only one specimen of *Kiwa hirsuta* (a male), was collected and its chelae – while stouter and broader than the afore mentioned species – appear to have similar, if not slightly less prominent, chela dentition, but teeth appear to be absent on *Kiwa tyleri* chelae. The presence of teeth on chelae may suggest some kiwaid diets are not exclusively microbial and *Kiwa hirsuta* individuals were observed eating bathymodiolid mussels, which had been damaged by the submersible (23), but it is unclear if they would have been capable of breaking into the mussels without assistance. The description of *Kiwa gemma* notes that the female chelae are more slender with less prominent teeth than the males, possibly indicating the cheliped may to some extent be also used in intraspecific competition between males(27).

As well as possible differences in chelae dentition between the sexes, the overall size and shape of the chelipeds between the sexes have been previously remarked upon. The large individuals of *Kiwa tyleri* collected from close to the vent orifice, all of which were males (Figure 3), were described as having longer and broader chelipeds (25,40,42) compared with smaller individuals collected further away, and interactions described as 'fighting' recorded between pairs of large individuals (42). Liu et al. (27) observed that amongst the 18 examined specimens of *Kiwa gemma*, the typically larger male specimens exhibited chelipeds around three times the length of the carapace, versus 1.6 times in females. To date only one study had attempted to explicitly explore sexual dimorphism in kiwaid chelae. Azofeifa-Solano et al. (43) measured more than 200 *Kiwa puravida* specimens comparing the length and breadth of the chela propodus vs carapace length, revealing the individuals with the largest chelae in proportion to the carapace to be male, which were also the largest individuals. Their analysis, however, assumed isometry with respect to chelae and carapace growth, which was found herein to not be the case, therefore precluding any inferences regarding sexual dimorphism when accounting for size.

Figure 3. Female (A) and male (B) *Kiwa tyleri* type specimens, modified from Thatje et al. (25). Scale bar is approximate and represents 10 mm.

Aims

This study aims to gain further insight into the function of kiwaid chelipeds by exploring the extent of chelae asymmetry in specimens of *Kiwa tyleri*, and to determine whether males have larger chelae than females when accounting for size, indicative of competition between males for access to females – i.e. sexual selection. For comparative purposes *Kiwa puravida* measurements from Azofeifa-Solano et al. (43) have also been re-analysed to determine any general patterns within Kiwaidae.

Materials & Methods

Sampling

Specimens of the yeti crab *Kiwa tyleri* were collected from hydrothermal vents on the E2 and E9 ridge segments of the East Scotia Ridge(25,35) (Figure 4), Southern Ocean on two separate NERC-funded expeditions aboard the *RRS James Cook* (JC042 – 07 January 2010 to 24 February 2010; JC080 – 02 December 2012 to 30 December 2010), part of the ChEsSO project. The E2 vent field is situated between 56°05.29' and 56°05.49'S and 30°19.00’ and 30°19.36'W at ~2600 m depth and the E9 vent field is ~450 km further South at ~2400 m depth, between 60°02.50' and 60°03.00'S and 29°59.00’ and 29°58.60'W (Fig 1). Ambient sea temperatures at the periphery of the vent fields ranged from 0°C to -1.3°C at E2 and E9 respectively, and maximum fluid temperatures emanating from black-smoker chimneys were recorded as 352.6 °C and 382.8 °C at E2 and E9 respectively (35). Kiwaids at the base of chimneys were typically in temperatures ranging from 2-12°C, although individuals on the chimney sides may have experienced higher temperatures in closer proximity to the vent orifice (42,49) . Individuals were sampled from

areas of pillow basalt with diffuse venting as well as from the base and sides of 'black-smoker' chimneys with a suction sampler deployed from the ROV *ISIS* on dives 130, 132, 134, 135, 189 (E2) and dives 140, 141 and 144 (E9). Kiwaids were not systematically or randomly sampled and different locations had distinct size cohorts with skewed sex ratios (36,42,49). Aboard *RRS James Cook*, recovered specimens were triaged in a 4°C temperature-controlled room and either preserved initially in formalin (5% formaldehyde and buffered seawater) before being preserved in 70% ethanol, or preserved in 99% molecular grade ethanol, or frozen at -80°C for subsequent analyses ashore.

Figure 4. Map of *Kiwa tyleri* sampling locations on the East Scotia Ridge in the Scotia Sea, modified from other sources (50–54). ESR = East Scotia Ridge. Areas shaded in the light grey are ≤3000 m depth. (B) (i) Shaded bathymetric contour map of the ESR and (ii) depth profile of the ESR axis, modified from other sources (51,55). In all Figure panels, triangles represent hydrothermal vents. Double lines in (A) and (B) (i) indicate actively spreading ridge.

Measurements

Kiwa tyleri individuals were sexed depending on the presence of gonopores on the first segment of the third pereopod, which is only exhibited in females. Body parts were measured using digital vernier callipers to an accuracy of 0.1 mm. Carapace length was measured from the base of the rostrum (defined as where the supraocular spine meets the rostrum) to the posterior dorsal margin of the carapace. Two segments of both right and left chelipeds were measured: the propodus, which is the largest component of the claw comprising the 'palm' (manus) and the 'fixed finger' (pollex), and the carpus – the proximal segment closer to the body medial line. For each segment, the length from the proximal to distil margins, along with height, as the maximum distance across the segment at a right angle to the length (Figure 5).

Figure 5. Measurements of the external morphology of *Kiwa tyleri*. CL = carapace length, CpL = cheliped carpus length, CpH = cheliped carpus height, PL = cheliped propodus length, PH = cheliped propodus height. Scale bar = 10 mm (1 cm).

Statistical Analyses

The non-random and non-systematic sampling of *Kiwa tyleri* individuals precludes any statistical analyses regarding size distributions or sex ratios. Propodus length and height symmetry in individuals with both chelipeds was assessed by calculating an asymmetry index (AI) according to Van Valen (56); AI = (Right - Left) / (Right + Left). Chi-square tests were performed to see if the proportion of left-dominant or right-dominant individuals deviated from a 1:1 ratio with respect to propodus length and height. After data were determined not to be normally distributed or exhibit equal variances, differences in propodus length and height AI between males and females were assessed with non-parametric Wilcoxon Rank Sum tests. As multiple tests (4) were performed to examine asymmetry, a simple Bonferroni correction was performed (adjusted alpha = 0.0125). For all subsequent body proportion analyses, the larger or dominant cheliped measurement was used. The hypotheses that some *Kiwa tyleri* body-part dimensions (dependent variables) are differently proportioned according to sex (predictor categorical variable) when controlling for carapace length (continuous predictor variable) were tested with linear models to determine the significance of any differences in slope or intercept between males and females. To meet linear model assumptions, scatter plots were first examined for linearity between the continuous predictor and dependent variables. Additionally, homoscedasticity and normality of residuals was assessed with residuals vs fitted values plots, and Q-Q plots. Non-linearity was found in all comparisons between cheliped dimensions and carapace length, with all cheliped dimensions becoming proportionally larger with increasing size, i.e. exhibiting positive allometric growth. Consequently, data were log-transformed ($\log_{10}$) to better meet model assumptions. Linear models were performed with an interaction term between the continuous predictor variable (carapace length) and the categorical predictor variable (sex) to test for differences in slope and offset (intercept) between sexes. If the slope (interaction) was not significant, a simpler model without the interaction term was run to explicitly test if the offset alone was significantly

different. As multiple (10) linear models were performed to detect size-controlled sexual dimorphism in chelipeds, a simple Bonferroni correction was performed (alpha = 0.005).

In addition to the *Kiwa tyleri* data acquired herein, published data from Azofeifa-Solano et al. (43) on *Kiwa puravida* carapace length vs propodus (claw) length and height (reported as width by the authors) were re-analysed according to the above methodology for comparative purposes. As with *Kiwa tyleri*, data were non-linear (positive allometry) and were therefore log-transformed. All statistical analyses and data visualisation was performed in R studio (57). We wish to thank the authors of Azofeifa-Solano et al. (43) for publishing the raw data, highlighting its importance for subsequent analyses by the scientific community, particularly in the field of deep-sea biology where access to specimens is limited.

Results

A total of 135 *Kiwa tyleri* specimens were measured, of which 109 had both chelipeds fully intact. 67 (49.6%) individuals measured were female and 68 (50.4%) were male. Carapace lengths ranged from 15.2 mm to 52.7 mm in females (mean = 29.4 mm) and from 13.7 to 72.5 mm in males (mean = 38.6 mm). Raw measurements are reported in Supplementary Information (Table S1) and mean appendage measurements are reported in Table 1. The largest 10 specimens were all male.

Table 1. Summary of *Kiwa tyleri* measurements including *Kiwa puravida* measurements extracted from Azofeifa-Solano et al. (43). Mean measurements are shown with ± standard deviation. N = number, CL = carapace length, PL = cheliped propodus length, PH = cheliped propodus height, CpL = cheliped carpus length, CpH = cheliped propodus height.

Species	Sex	N	CL (mm)	PL (mm)	PH (mm)	CpL (mm)	CpH (mm)
Kiwa tyleri	Female	67	29.4 ± 9.37	13.9 ± 5.08	8.1 ± 2.98	9 ± 3.43	8.4 ± 3.02
	Male	68	38.6 ± 14.32	21.7 ± 10.69	13.2 ± 5.98	14.7 ± 7.33	13.4 ± 5.78
	Total	135	34.1 ± 12.93	17.8 ± 9.22	10.7 ± 5.39	11.9 ± 6.39	10.9 ± 5.25
Kiwa puravida	Female	68	8 ± 6.12	5.3 ± 4.65	1.8 ± 1.48	NA	NA
	Male	138	6.6 ± 7.32	4.6 ± 6.30	1.5 ± 1.98	NA	NA
	Total	206	7 ± 6.97	4.8 ± 5.81	1.6 ± 1.83	NA	NA

For the propodus asymmetry index (AI), negative values indicate a left skew and positive values a right skew. Of the 109 specimens with two chelipeds, 17 exhibited no measurable deviation in symmetry (AI = 0) for propodus length, with 54 left dominant and 34 right dominant. 21 exhibited no symmetry deviations for propodus height, with 38 left dominant and 49 right dominant. Mean AI values were close to 0, with -0.0057 (standard deviation = 0.024) for propodus length and -0.00059 (standard deviation = 0.0268) for propodus height (Figure 6). The maximum deviation from symmetry for length was 0.181 i.e., where the left propodus was 36.2% longer than the right propodus. The maximum symmetry deviation for propodus height was 0.153, where the left propodus was 30.6% broader than the right propodus. Both these extreme asymmetries were found in the same individual. After Bonferroni correction (alpha = 0.0125), Wilcoxon Rank Sum tests revealed no significant difference in AI between males and females for

propodus length (W = 1346, p = 0.410) and height (W = 1269, p = 0.250). Chi-square also revealed no significant deviation from 1:1 left-right dominance with respect to propodus length (chi-square = 4.546, df = 1, p-value = 0.033) or height (chi-square = 1.391, df = 1, p-value = 0.238).

Figure 6. Box and whisker plot showing asymmetry index (AI) values for cheliped propodus length and height in 109 specimens of *Kiwa tyleri*. Red dot = mean; thick horizonal line = median. The box represents the interquartile range (middle 50% of the data) and whiskers extend 1.5 * interquartile range from the lower and upper quartiles, respectively. Black dots represent outliers (outside 1.5 * interquartile range).

Cheliped propodus and carpus lengths and heights for *Kiwa tyleri* did not scale linearly with carapace length (Figures 7 and 8 X). In all cases, the relative size of the cheliped dimension increased in relation to carapace size as it increased, as is the case with the *Kiwa puravida* cheliped propodus dimensions taken from Azofeifa-Solano et al. (43) (Figure 9). Linear models of log-transformed measurements were all highly significant with p-values below 2.2×10^{-16} and adjusted R^2 values over 0.98, indicating at least 98% of the cheliped dimension was explained by the predictor variables (Supplementary Information Table S2).

For *Kiwa tyleri*, linear models of log-transformed carapace length vs cheliped dimensions indicated significant differences between males and females (Table 2). After Bonferroni correction (alpha = 0.005), there was a significant interaction between sex and log carapace length on log propodus length ($p = 4.20 \times 10^{-07}$) and log carpus length ($p = 1.42 \times 10^{-04}$) with a steeper slope for males (Figures 7 and 8), i.e. male cheliped lengths becoming proportionately larger than females with increasing carapace length, indicative of faster cheliped growth rates. For log propodus height (p = 0.023) and log carpus height (p = 0.0969), there was no significant difference in the angle of slope between males and females, but there was a highly significant difference in the y-axis offset in the simpler models without the interaction term ($p < 2 \times 10^{-16}$) (Table 2) with males having a broader log carpus and propodus heights than females throughout

the size distribution of log carapace lengths (Figures 7 and 8). Given the differences in positive allometry with respect to cheliped lengths and heights, as males grow in size their chelipeds become more slender in shape at the upper end of the size spectrum. Conversely, linear models of the log-transformed dataset for *Kiwa puravida* from Azofeifa-Solano et al. (43) showed no effect of sex on slope for log propodus length (p = 0.834) or height (p = 0.45) vs log carapace length, and no effect of sex on y-axis offset in the simpler models without the interaction term (log propodus length, p = 0.371; log propodus height, p = 0.473) (Figure 9, Table 2).

Table 2. Linear model predictor coefficient output regarding the effect of log carapace length and sex (continuous and categorical predictor variables respectively) – or the interaction between the two – on log cheliped dimensions in the yeti crab species *Kiwa tyleri* and *Kiwa puravida*. Ten models were run. Initially models included interaction between log carapace length and sex, and if non-significant, a simpler model without the interaction term was run. Bonferroni corrections were performed, with significance below 0.005 designated with an asterisk. Model predictor terms of interest in the study are highlighted in bold. B = the coefficient estimate for each predictor variable. Std. Error = the standard error of the estimate. t = the t-statistic, calculated as the estimate divided by the standard error. P = P-value, indicating significance level of the predictor term in the model. For complete linear model outputs, see Table S2 in Supplementary Information.

Species	Dependent Variable	Model predictor terms	B	Std. Error	t	P	Sig.
Kiwa tyleri	Log propodus length	Log carapace length	1.117	0.023	48.756	0.000	*
		Sex	-0.198	0.044	-4.478	0.000	*
		**Log carapace length : Sex**	**0.157**	**0.030**	**5.328**	**0.000**	*
	Log propodus height	Log carapace length	1.144	0.024	47.138	0.000	*
		Sex	-0.038	0.047	-0.803	0.423	
		**Log carapace length : Sex**	**0.072**	**0.031**	**2.303**	**0.023**	
	Log propodus height	Log carapace length	1.187	0.016	76.320	0.000	*
		**Sex**	**0.070**	**0.005**	**13.750**	**0.000**	*
	Log carpus length	Log carapace length	1.174	0.025	47.660	0.000	*
		Sex	-0.131	0.048	-2.750	0.007	
		**Log carapace length : Sex**	**0.124**	**0.032**	**3.919**	**0.000**	*
	Log carpus height	Log carapace length	1.117	0.022	49.733	0.000	*
		Sex	-0.007	0.043	-0.152	0.880	
		**Log carapace length : Sex**	**0.048**	**0.029**	**1.672**	**0.097**	
	Log carpus height	Log carapace length	1.146	0.014	80.340	0.000	*
		**Sex**	**0.066**	**0.005**	**14.100**	**0.000**	*
Kiwa puravida	Log propodus length	Log carapace length	1.082	0.017	63.183	0.000	*
		Sex	0.013	0.017	0.769	0.443	
		**Log carapace length : Sex**	**-0.004**	**0.021**	**-0.210**	**0.834**	
	Log propodus length	Log carapace length	1.079	0.010	111.674	0.000	*
		**Sex**	**0.011**	**0.012**	**0.897**	**0.371**	
	Log propodus height	Log carapace length	1.065	0.017	61.024	0.000	*
		Sex	-0.018	0.018	-1.043	0.298	
		**Log carapace length : Sex**	**0.016**	**0.021**	**0.757**	**0.450**	
	Log propodus height	Log carapace length	1.076	0.010	109.079	0.000	*
		**Sex**	**-0.009**	**0.012**	**-0.718**	**0.473**	

Figure 7. Scatter plots of dominant cheliped propodus dimensions for 135 specimens of *Kiwa tyleri* collected from from hydrothermal vents on the East Scotia Ridge in the Southern ocean. A) propodus length vs carapace length and B) log-transformed data. C) propodus height vs carapace length and D) log-transformed data. Blue dots are females and orange triangles are males. Lines in B) and D) represent linear models fitted to the data, with adjadent shaded areas showing 95% confidence interval as calculated in R.

Figure 8. Scatter plots of dominant cheliped carpus dimensions for 135 specimens of *Kiwa tyleri* collected from from hydrothermal vents on the East Scotia Ridge in the Southern ocean. A) carpus length vs carapace length and B) log-transformed data. C) carpus height vs carapace length and D) log-transformed data. Blue dots are females and orange

triangles are males. Lines in B) and D) represent linear models fitted to the data, with adjadent shaded areas showing 95% confidence interval as calculated in R.

Figure 9. Scatter plots of cheliped propodus dimensions for 206 specimens of *Kiwa puravida* taken from Azofeifa-Solano et al. (43). A) propodus length vs carapace length and B) log-transformed data. C) propodus height vs carapace length and D) log-transformed data. Blue circles are females and orange hollow triangles are males. Lines in B) and D) represent linear models fitted to the data, with adjadent shaded areas showing 95% confidence interval as calculated in R.

Discussion

Claw symmetry

This is the first exploration of cheliped symmetry in yeti crabs (Kiwaidae), indicating that *Kiwa tyleri* exhibits a high degree of symmetry in cheliped propodus dimensions, with asymmetry indices (AI) for most individuals close to zero, no significant differences in AI between sexes, or deviations from 1:1 ratio for left and right dominance. There were some outliers, including an individual whose left cheliped propodus was over 30% larger than the right, which could be explained by either a developmental asymmetry, disease, parasitism, or the consequence of damage and/or cheliped loss and subsequent regrowth, as reported for some decapods (45). There is no evidence of any of the specimens in this study having lost a cheliped prior to sampling, however, and loose chelipeds were found at the bottom of the containers in which specimens were batch-preserved, suggesting that they detached after specimen triage.

The high degree of propodus symmetry in *Kiwa tyleri* may provide insights into cheliped function in this species. Asymmetry (heterochely) in decapod chelipeds is generally associated with specialisation relating to prey handling/manipulation, sound generation, or in some cases sexually-selected activity e.g. display in fiddler crabs or conspecific fighting in (male and female) aeglid squat lobsters (45,58–60), although there is continued debate regarding the links between ecology and heterochely (48). The bythograeid crab *Austingraea williamsi*, found at vents in the Mariana Back-Arc Basin often exhibits larger crusher and more slender 'cutter' chelae commonly observed in other brachyuran predators of shelled organisms (46). The lack of heterochely in *Kiwa tyleri* may therefore be consistent with chelipeds being used primarily for functions other than carnivory, such as microbial food production and/or intraspecific agonistic interactions. The primary reliance of *Kiwa tyleri* on farming microbes for sustenance is indicated by *in situ*

observation, morphology of feeding appendages and setae, and analyses of $\partial^{13}C$, $\partial^{15}N$ and $\partial^{34}S$ values indicating chemosynthetically-derived nutrition (32,25,31).

Spani et al. (48) found a strong phylogenetic signal with heterochely, which suggests that cheliped symmetry may be characteristic of Kiwaidae, but examination of the other species within the family will be necessary to confirm this. To date, we are unaware of heterochely being investigated in the other two sister families within the superfamily Chirostyloidea: Chirostylidae and Eumunididae. The feeding ecology of these two deep-water coral-associated families is presently unclear, although some observations suggest they may use corals as platforms for collecting suspended food particles or catching pelagic mesofauna (61–63), rather than the durophagy often associated with Brachyura (45). Both families exhibit prominently elongated chelipeds, similar to the yeti crab bauplan, and it may be that this mode of feeding in Chirostylidae and Eumunididae is ancestral for the superfamily based on phylogeny (26), even though the only-known stem-lineage kiwaid fossil was not found in association with coral deposits (64).

Cheliped growth and sexual dimorphism

The results of the linear models clearly indicate that when controlling for size, *Kiwa tyleri* males exhibited faster growth rates for cheliped propodus and carpus lengths (~~greater~~ positive allometry) and throughout the size distribution had generally broader and longer chelipeds, than females. In stark contrast, *Kiwa puravida* individuals did not show any differences in positive allometry with respect to sex, or any differences in cheliped proportions for individuals of similar size. The reanalyses of *Kiwa puravida* data contradicts Azofeifa-Solano et al. (43), which can be attributed to the use of log transformation in this study. Cheliped measurements for *Kiwa tyleri* and *Kiwa puravida* did not scale linearly with carapace length (Figures 7, 8 and 9) instead showing positive allometry, necessitating the use of log-transformation to better fit linear model assumptions. It appears therefore that the apparent difference in linear regressions between

males and females in that study was due to the unequal size distribution between the sexes, with the largest individuals being male, as they are in this study.

This is the first quantitative morphological study to show differences in yeti crab cheliped dimensions and growth rates between males and females when controlling for size. The difference in body shape was previously observed in *Kiwa tyleri* (35,36,42,49), but the size difference between males and female specimens was not accounted for (Figure 3) and while this difference may reflect sexual selection relating to male-male competition for females, it could also reflect limitations on female growth owing to the constraints of brooding eggs further away from anoxic vent/seep effluent; thereby limiting access to microbial sustenance (40). A food-limitation hypothesis for smaller female size is supported by the observation that brooding female *Kiwa tyleri* individuals collected at the periphery of vents were typically rust-coloured (presumably as a result of oxidation of iron compounds deposited on the exoskeleton), indicative of a larger elapsed time since moulting compared with individuals collected closer to the vents (40). The differences in cheliped size for similarly sized *Kiwa tyleri* males and females found in this study is the strongest indication yet of sexual selection in Kiwaidae relating to male-male competition for mating access, consistent with observations of individuals 'fighting' (24,42).

Larger cheliped weaponry in males vs similarly-sized females is widely reported in decapod crustaceans (45,65,66) and is typically associated with either agonistic male-male interactions or visual display for access to females. Given the apparent absence of functioning eyes in Kiwaidae, the possibility of cheliped sexual dimorphism for visual display purposes is remote. Asakura et al (65) listed several mating systems in decapod crustaceans, but the 'precopulatory guarding' type where males resource guard a female for a period to time prior to mating, arising when male-male competition for females intense, and/or the window for mating with a receptive female is time constrained, is more typical in anomuran squat lobsters (66). In squat lobsters, the male typically embraces the female dorsally, holding her in place by grasping with the cheliped or

pereopods (66) and Thatje and Marsh (42) observed that *Kiwa tyleri* males exhibited a substantially-enlarged "comb of strong, flattened, teeth-like spines" on the ischium to carpus cheliped segments which would allow the male to "arrest the female on its lateral margins of the carapace". If their interpretation is correct, this would suggest that *Kiwa tyleri* conforms to the precopulatory mating type, as Azofeifa-Solano et al. (43) concluded with *Kiwa puravida*.

The absence, however, of apparent cheliped sexual dimorphism when controlling for size in *Kiwa puravida* does warrant explanation. One possibility could relate to differences in size between *Kiwa tyleri* and *Kiwa puravida* ~~in the study.~~ Reviewing sexual dimorphism in squat lobsters, Thiel & Lovrich (66) noted that in larger species, males tend to become sexually mature at smaller sizes than females compared with smaller species where males and females reach maturity at similar sizes. *Kiwa tyleri* appears generally much larger than *Kiwa puravida*, with the largest *Kiwa puravida* individual reported by Azofeifa-Solano et al. (43) (carapace length = 29.4mm) being far smaller than the largest *Kiwa tyleri* individual (72.5 mm) reported here. *K. tyleri* males may reach sexual maturity at smaller sizes in their size distribution, exhibiting sexual dimorphism comparatively earlier. If *Kiwa puravida* males reach sexual maturity only at the very upper end of their size distribution, then any detection of differences in positive allometry would hypothetically be reliant on measuring sufficient numbers of as-yet unsampled larger males exhibiting disproportionately larger chelipeds. However, no such *Kiwa puravida* individuals have been reported thus far (24,43). In contrast with *Kiwa puravida*, even the smallest individuals of *Kiwa tyleri* exhibited cheliped proportion differences between the sexes suggesting an ecological difference between the two species.

Kiwa tyleri, and *Kiwa* sp. SWIR are notable amongst kiwaids in having the preponderance of their setae on their ventral surface, rather than on elongated chelipeds. It may be therefore that the use of the chelipeds as a principal surface for farming microbes in *Kiwa puravida* (and possibly other elongated kiwaids) places an evolutionary constraint on the sexual selection for oversized male

chelipeds in those taxa. Thurber et al. (24) reported *Kiwa puravida* individuals waving their chelipeds back and forth in a rhythmic fashion, which is a behaviour not seen in other kiwaids. The inference is that *Kiwa puravida*, inhabiting low-flow methane seeps wave their chelipeds to enhance fluid flow and boost microbial productivity, suggesting their chelipeds are critical for food production. In contrast, *Kiwa tyleri* may be less reliant on chelipeds for sustenance, not only due to the location of their setae, but also if the vents they inhabit engender greater microbial production (as evidenced by their considerably larger maximum body size), thus freeing chelipeds for other functions, including as sexually selected weapons in male-male agonistic interactions. *Kiwa tyleri* are also far more numerically dominant than *Kiwa puravida*, often existing in very high densities (36,67) and the difference in cheliped sexual dimorphism between the two species may reflect more intensive competition between *Kiwa tyleri* individuals inhabiting a narrow viable thermal envelope around the Southern Ocean vents, as is shown for cave-dwelling munidopsid squat lobsters (68).

If the prevalence and intensity of male-male competition is similar between *Kiwa puravida* and *Kiwa tyleri*, it is also possible that *Kiwa puravida* exhibits sexually selected dimorphism in body parts as yet unmeasured, such as the cheliped carpus. It would also be interesting to see if *Kiwa puravida* males have enlarged comb-like spines on the proximal cheliped segments as observed by Thatje and Marsh (42) in *Kiwa tyleri*, and indeed, if all the spines on the chelipeds and/or the teeth on the cutting edge of the chela are enlarged. Calverie and Smith (69) observed male munidid squat lobsters exhibiting more prominent teeth on the chela dactylus (claw finger) suggesting their use for puncturing the chelipeds of male rivals and preliminary observations hint this may be the case with *Kiwa gemma*, which belongs to the same bristly clade as *Kiwa puravida* (27). It is notable in this context that *Kiwa tyleri* appears to lack a prominent chela tooth, unlike all three bristly species, with *Kiwa hirsuta* intermediate (23–27). If cheliped dentition in *Kiwa puravida* is sexually dimorphic, it could suggest that different constraints on cheliped function between *Kiwa puravida* and *Kiwa tyleri* determines the evolutionary avenue for the development

of male-male agonistic weaponry, or that the nature of male-male interactions are fundamentally different (e.g. pushing and sparring vs. puncturing) owing to other ecological factors (e.g. population density). One way to explore this further would be to see if *Kiwa puravida* males exhibit more puncture wounds than females alongside analyses of footage to better characterise agonistic interactions in both species.

Based on the results presented in this study, it is unclear whether – or in what way – cheliped sexual dimorphism is prevalent in the other kiwaids. Given the elongated chelipeds of *Kiwa hirsuta*, *Kiwa gemma* and *Kiwa araonae*, possibly indicating their primary use as platforms for microbial farming, cheliped sexual dimorphism throughout the size distribution may be absent or limited to more subtle features like chela shape and tooth architecture. Alternatively, *Kiwa puravida* may be the exception, inhabiting low-flow sites around methane seeps where reduced microbial productivity necessitates prioritising waving behaviour over agonistic behaviour, constraining the potential scope for sexually selected cheliped dimorphism.

Study limitations and future direction

This study attempts to make inferences about the feeding and reproductive ecology of yeti crabs based primarily on morphology. However, these inferences would need to be confirmed by either prolonged *in situ* observation in the deep sea and subsequent specimen collection to confirm sex (a challenge owing to the remoteness of deep-sea chemosynthetic habitats), or through observation in aquaria, which may be feasible in *Kiwa puravida* where some individuals have been kept alive for several months in aquaria (43), but has yet to be demonstrated with *Kiwa tyleri*, or other yet crabs which inhabit far greater depths. (> 1800 m). The sampling of *Kiwa tyleri* in this study was neither systematic nor random, precluding some population inferences about size distribution. In the future a random or systematic sampling approach might better determine if the largest individuals are indeed male as current and previous studies suggest for

these species (and other kiwaids), although there are ethical considerations with over sampling species endemic to isolated locations. The question of whether *Kiwa tyleri* or *Kiwa puravida* represents the more basal or derived state for cheliped dimorphism can only be determined by the additional sampling of the other kiwaid species, including *Kiwa hirsuta* where only one specimen was ever sampled (23). However, opportunities for sampling kiwaids are extremely limited as mid-ocean ridges are very remote. For example, as far as we are aware, the hydrothermal vents on the Pacific-Antarctic Ridge in the SE Pacific have not been sampled since 2005 and vents on the East Scotia Ridge in the Southern Ocean since 2012.

Conclusion

This is the first morphological study to investigate cheliped symmetry in Kiwaidae, and cheliped allometry and sexual dimorphism in *Kiwa tyleri*. While size differences between male and female yeti crabs have been previously reported, the results for *Kiwa tyleri* presented in this study are the first to show differences in cheliped proportions between male and female kiwaids of a similar size, with males exhibiting proportionally larger chelipeds, indicative of sexual selection for larger weaponry in male-male competition for reproductive access to females. Conversely, *Kiwa puravida* individuals do not display this form of dimorphism, reflecting either differences in mating system, in the nature and intensity of male-male competition, and/or in the functional use of the chelipeds between the two species. The extent of sexual dimorphism in the other kiwaid species is unclear, but this study represents an early step in elucidating the basic ecology of the yeti crabs, which is critical if highly endemic, geographically isolated species such as these are to be protected from present and future anthropogenic threats.

Acknowledgements

We wish to thank the crew and scientists aboard the two *RRS James Cook* expeditions (JC042, JC080), funded by NERC Grant NE/D01249X/1, for the collection of the *Kiwa tyleri* specimens and the technicians at the Institute of Marine Science in Portsmouth, UK for their support in the lab. This study would also not have been possible without the initial work by Dr Sven Thatje and Vittoria Francis. Additionally, we thank the authors of Azofeifa-Solana et al. (43) for publishing their raw data which was invaluable for this study.

Author contributions

C. Nicolai Roterman

Roles: Conceptualisation, Specimen collection, Data curation, Formal analysis, Investigation, Methodology, Project administration, Supervision, Validation, Visualisation, Writing – original draft, Writing – review & editing.

* E-mail: nicolai.roterman@port.ac.uk

Affiliation: Institute of Marine Science (IMS), University of Portsmouth, Portsmouth, PO4 9LY, UK

ORCID: https://orcid.org/0000-0002-6636-6078

Molly McArthur

Roles: Data curation, Formal analysis, Methodology, Writing – review & editing.

Affiliation: Institute of Marine Science (IMS), University of Portsmouth, Portsmouth, PO4 9LY, UK

Cecilia Laverty-Baralle

Roles: Data curation, Formal analysis, Methodology, Writing – review & editing.

Affiliation: Institute of Marine Science (IMS), University of Portsmouth, Portsmouth, PO4 9LY, UK

Leigh Marsh

Roles: Conceptualisation, Specimen Collection, Methodology, Writing – review & editing.

Affiliation: Ocean and Earth Science, University of Southampton, Waterfront Campus, Southampton SO14 3ZH, UK

ORCID: https://orcid.org/0000-0002-4613-1538

Jon Copley

Roles: Conceptualisation, Specimen Collection, Funding, Methodology, Project Administration, Writing – original draft, Writing, review & editing.

Affiliation: Ocean and Earth Science, University of Southampton, Waterfront Campus, Southampton SO14 3ZH, UK

ORCID: https://orcid.org/0000-0003-3333-4325

Funding

This research was supported by a NERC (UK) Consortium Grant (NE/D01249X/1; ChEsSo) and publication was funded by the University of Portsmouth.

References

1. Levin LA, Baco AR, Bowden DA, Colaço A, Cordes EE, Cunha MR, et al. Hydrothermal Vents and Methane Seeps: Rethinking the Sphere of Influence. Front Mar Sci. 2016 May 19;3(e76869):580.

2. Corliss JB, Dymond J, Gordon LI, Edmond JM, Herzen RPV, Ballard RD, et al. Submarine thermal springs on the Galapagos Rift. Science. 1979;203(4385):1073–83.

3. Decelle J, Andersen AC, Hourdez S. Morphological adaptations to chronic hypoxia in deep-sea decapod crustaceans from hydrothermal vents and cold seeps. Mar Biol. 2010;157(6):1259–69.

4. Hourdez S, Lallier FH. Adaptations to hypoxia in hydrothermal-vent and cold-seep invertebrates. Rev Environ Sci Biotechnol. 2007;6(1–3):143–59.

5. Bris NL, Gaill F. How does the annelid Alvinella pompejana deal with an extreme hydrothermal environment? Rev Environ Sci Biotechnol. 2006;6(1–3):197.

6. Cottin D, Shillito B, Chertemps T, Thatje S, Léger N, Ravaux J. Comparison of heat-shock responses between the hydrothermal vent shrimp Rimicaris exoculata and the related coastal shrimp Palaemonetes varians. J Exp Mar Biol Ecol. 2010;393(1–2):9–16.

7. Ravaux J, Hamel G, Zbinden M, Tasiemski AA, Boutet I, Léger N, et al. Thermal Limit for Metazoan Life in Question: In Vivo Heat Tolerance of the Pompeii Worm. PLoS ONE. 2013;8(5):e64074.

8. Ravaux J, Léger N, Hamel G, Shillito B. Assessing a species thermal tolerance through a multiparameter approach: the case study of the deep-sea hydrothermal vent shrimp Rimicaris exoculata. Cell Stress Chaperones. 2019 May 9;24(3):647–59.

9. Gaill F, Bouligand Y. Supercoil of collagen fibrils in the integument of *Alvinella*, an abyssal annelid. Tissue Cell. 1987 Jan 1;19(5):625–42.

10. Yao HM, Dao M, Imholt T, Huang JM, Wheeler K, Bonilla A, et al. Protection mechanisms of the iron-plated armor of a deep-sea hydrothermal vent gastropod. Proc Natl Acad Sci. 2010;107(3):987–92.

11. Machon J, Krieger J, Meth R, Zbinden M, Ravaux J, Montagné N, et al. Neuroanatomy of a hydrothermal vent shrimp provides insights into the evolution of crustacean integrative brain centers. eLife. 2019;8:e47550.

12. O'Neill PJ, Jinks RN, Herzog ED, Battelle BA, Kass L, Renninger GH, et al. The morphology of the dorsal eye of the hydrothermal vent shrimp, Rimicaris exoculata. Vis Neurosci. 1995;12(05):861–75.

13. Yancey PH, Blake WR, Conley J. Unusual organic osmolytes in deep-sea animals: adaptations to hydrostatic pressure and other perturbants. Comp Biochem Physiol A Mol Integr Physiol. 2002 Nov 1;133(3):667–76.

14. Yancey PH, Ishikawa J, Meyer B, Girguis PR, Lee RW. Thiotaurine and hypotaurine contents in hydrothermal-vent polychaetes without thiotrophic endosymbionts: correlation With sulfide exposure. J Exp Zool Part Ecol Genet Physiol. 2009;311A(6):439–47.

15. Sogin EM, Kleiner M, Borowski C, Gruber-Vodicka HR, Dubilier N. Life in the Dark: Phylogenetic and Physiological Diversity of Chemosynthetic Symbioses. Annu Rev Microbiol. 2021 Oct 8;75(1):695–718.

16. Goffredi SK. Indigenous ectosymbiotic bacteria associated with diverse hydrothermal vent invertebrates. Environ Microbiol Rep. 2010 Aug;2(4):479–88.

17. Zbinden M, Cambon-Bonavita M. Rimicaris exoculata: biology and ecology of a shrimp from deep-sea hydrothermal vents associated with ectosymbiotic bacteria [Internet]. 2020. Available from: https://www.int-res.com/articles/meps_oa/m652p187.pdf

18. Watanabe HK, Chen C, Chan BKK. A new deep-sea hot vent stalked barnacle from the Mariana Trough with notes on the feeding ecology of Vulcanolepas. Mar Biodivers. 2021 Feb 1;51(1):1–12.

19. Duperron S, Lorion J, Samadi S, Gros O, Gaill F. Symbioses between deep-sea mussels (Mytilidae: Bathymodiolinae) and chemosynthetic bacteria: diversity, function and evolution. C R Biol. 2009;332(2–3):298–310.

20. Hinzke T, Kleiner M, Breusing C, Felbeck H, Häsler R, Sievert SM, et al. Host-Microbe Interactions in the Chemosynthetic Riftia pachyptila Symbiosis. mBio. 2019;10(6):e02243-19.

21. Chen C, Uematsu K, Linse K, Sigwart JD. By more ways than one: Rapid convergence at hydrothermal vents shown by 3D anatomical reconstruction of Gigantopelta (Mollusca: Neomphalina). BMC Evol Biol. 2017 Mar 1;17(1):62.

22. Chen C, Linse K, Uematsu K, Sigwart JD. Cryptic niche switching in a chemosymbiotic gastropod. Proc R Soc B Biol Sci. 2018 Jul 11;285(1882):20181099–138.

23. Macpherson E, Jones W, Segonzac M. A new squat lobster family of Galatheoidea (Crustacea, Decapoda, Anomura) from the hydrothermal vents of the Pacific-Antarctic Ridge. Zoosystema. 2005;27(4):709–23.

24. Thurber AR, Jones WJ, Schnabel K. Dancing for Food in the Deep Sea: Bacterial Farming by a New Species of Yeti Crab. PLoS ONE. 2011;6(11):e26243.

25. Thatje S, Marsh L, Roterman CN, Mavrogordato MN, Linse K. Adaptations to Hydrothermal Vent Life in Kiwa tyleri, a New Species of Yeti Crab from the East Scotia Ridge, Antarctica. Steffen Kiel, editor. Plos One. 2015 Jun 24;10(6):e0127621.

26. Lee SH, Lee WK, Won YJ. A new species of yeti crab, genus Kiwa Macpherson, Jones and Segonzac, 2005 (Decapoda: Anomura: Kiwaidae), from a hydrothermal vent on the Australian-Antarctic Ridge. J Crustac Biol. 2016 Feb 23;36(2):238–47.

27. Liu X, Lin J, Mendoza JCE. A new species of the bathyal yeti crab genus Kiwa Macpherson, Jones & Segonzac, 2005 (Decapoda: Anomura: Kiwaidae) from a hydrothermal vent field in the Eastern Pacific Ocean, with a key to the genus. J Crustac Biol. 2024 Jun 1;44(2):ruae030.

28. Roterman CN, Lee WK, Liu X, Lin R, Li X, Won YJ. A new yeti crab phylogeny: Vent origins with indications of regional extinction in the East Pacific. Geerat J Vermeij, editor. Plos One. 2018 Mar 16;13(3):e0194696.

29. Roterman CN, Copley JT, Linse KT, Tyler PA, Rogers AD. The biogeography of the yeti crabs (Kiwaidae) with notes on the phylogeny of the Chirostyloidea (Decapoda: Anomura). Proc R Soc B Biol Sci. 2013 Aug 7;280(1764):20130718.

30. Goffredi SK, Jones WJ, Erhlich H, Springer A, Vrijenhoek RC. Epibiotic bacteria associated with the recently discovered yeti crab, Kiwa hirsuta. Environ Microbiol. 2008 Oct;10(10):2623–34.

31. Zwirglmaier K, Reid WDK, Heywood J, Sweeting CJ, Wigham BD, Polunin NVC, et al. Linking regional variation of epibiotic bacterial diversity and trophic ecology in a new species of Kiwaidae (Decapoda, Anomura) from East Scotia Ridge (Antarctica) hydrothermal vents. MicrobiologyOpen. 2015 Feb 1;4(1):136–50.

32. Reid WDK, Sweeting CJ, Wigham BD, Zwirglmaier K, Hawkes JA, McGill RAR, et al. Spatial differences in East Scotia Ridge hydrothermal vent food webs: influences of chemistry, microbiology and predation on trophodynamics. Plos One. 2013;8(6):e65553.

33. Reid W, Sweeting CJ, Wigham BD, McGill R, Polunin N. Isotopic niche variability in macroconsumers of the East Scotia Ridge (Southern Ocean) hydrothermal vents: what more can we learn from an ellipse? Mar Ecol-Prog Ser [Internet]. 2015; Available from: http://www.int-res.com/abstracts/meps/v542/p13-24/

34. Reid WDK, Wigham BD, Marsh L, Weston JNJ, Zhu Y, Copley JT. Trophodynamics at the Longqi hydrothermal vent field and comparison with the East Scotia and Central Indian Ridges. Mar Biol. 2020 Oct 1;167(10):1–16.

35. Rogers AD, Tyler PA, Connelly DP, Copley JT, James R, Larter RD, et al. The discovery of new deep-sea hydrothermal vent communities in the Southern Ocean and implications for biogeography. Plos Biol. 2012;10(1):e1001234.

36. Marsh L, Copley JT, Huvenne VAI, Linse K, Reid WDK, Rogers AD, et al. Microdistribution of faunal assemblages at deep-sea hydrothermal vents in the Southern Ocean. Plos One. 2012;7(10):e48348.

37. Frederich M, Sartoris FJ, Portner HO. Distribution patterns of decapod crustaceans in polar areas: a result of magnesium regulation? Polar Biol. 2001 Oct;24(10):719–23.

38. Aronson RB, Frederich M, Price R, Thatje S. Prospects for the return of shell-crushing crabs to Antarctica. J Biogeogr. 2015;42(1):1–7.

39. Thatje S, Smith KE, McClintock JB, Aronson RB. From deep to shallow seas: Antarctic king crab on the move. Ecology. 2020;101(11):e03125.

40. Marsh L, Copley JT, Tyler PA, Thatje S. In hot and cold water: differential life-history traits are key to success in contrasting thermal deep-sea environments. Tom Webb, editor. J Anim Ecol. 2015 Jul 1;84(4):898–913.

41. Thatje S, Smith KE, Marsh L, Tyler PA. Evidence for abbreviated and lecithotrophic larval development in the yeti crab Kiwa tyleri from hydrothermal vents of the East Scotia Ridge, Southern Ocean. Sex Early Dev Aquat Org. 2015;1(2):109–16.

42. Thatje S, Marsh L. From hot waters of polar seas: the mysterious life of the male yeti crab. Ecology. 2018 Aug 15;42(4):1.

43. Azofeifa-Solano JC, Pereira OS, Cowell EJ, Cordes EE, Levin LA, Goffredi SK, et al. Sexual dimorphism in the methane seep-dwelling Costa Rican yeti crab Kiwa puravida (Decapoda: Anomura: Kiwaidae). Front Mar Sci [Internet]. 2022 [cited 2023 Jun 21];9. Available from: https://www.frontiersin.org/articles/10.3389/fmars.2022.1051590

44. Shuker DM, Kvarnemo C. The definition of sexual selection. Behav Ecol. 2021 Sep 1;32(5):781–94.

45. Mariappan P, Balasundaram C, Schmitz B. Decapod crustacean chelipeds: an overview. J Biosci. 2000 Sep 1;25(3):301–13.

46. Tsuchida S, Fujikura K. Heterochely, Relative Growth, and Gonopod Morphology in the Bythograeid Crab, Austinograea Williamsi (Decapoda, Brachyura). J Crustac Biol. 2000 Mar 1;20(2):407–14.

47. Juanes F, Lee KT, McKnight A, Kellogg K. Claw allometry in green crabs, Carcinus maenas: heterochely, handedness, and sex. Mar Biol. 2008 Feb 1;153(4):523–8.

48. Spani F, Scalici M, Crandall KA, Piras P. Claw asymmetry in crabs: approaching an old issue from a new point of view. Biol J Linn Soc. 2020 Jan 1;129(1):162–76.

49. Marsh L, Copley JT, Tyler PA, Thatje S. In hot and cold water: differential life-history traits are key to success in contrasting thermal deep-sea environments. Tom Webb, editor. J Anim Ecol. 2015 Jul 1;84(4):898–913.

50. Roterman CN, Copley JT, Linse KT, Tyler PA, Rogers AD. Connectivity in the cold: the comparative population genetics of vent-endemic fauna in the Scotia Sea, Southern Ocean. Mol Ecol. 2016 Mar 1;25(5):1073–88.

51. Livermore R. The East Scotia Sea: mantle to microbe. Geophys Monogr. 2006;166:243–61.

52. Orsi AH, Johnson GC, Bullister JL. Circulation, mixing, and production of Antarctic Bottom Water. Prog Oceanogr. 1999 Jan;43(1):55–109.

53. Naveira-Garabato AC, Heywood KJ, Stevens DP. Modification and pathways of Southern Ocean deep waters in the Scotia Sea. Deep Sea Res Part Oceanogr Res Pap. 2002;49(4):681–705.

54. Meredith MP, Garabato ACN, Gordon AL, Johnson GC. Evolution of the deep and bottom waters of the Scotia Sea, Southern Ocean, during 1995-2005*. J Clim. 2008;21(13):3327–43.

55. Amon DJ, Glover AG, Wiklund H, Marsh L, Linse K, Rogers AD, et al. The discovery of a natural whale fall in the Antarctic deep sea. Deep Sea Res Part II Top Stud Oceanogr. 2013;92:87–96.

56. Van Valen L. A Study of Fluctuating Asymmetry. Evolution. 1962;16(2):125–42.

57. R Studio Team. RStudio: Integrated Development for R. RStudio, PBC, Boston, MA [Internet]. 2020. Available from: http://www.rstudio.com/.

58. Trevisan A, Santos S. Morphological Sexual Maturity, Sexual Dimorphism and Heterochely in Aegla Manuinflata (Anomura). J Crustac Biol. 2012 Jul 1;32(4):519–27.

59. Palaoro AV, Dalosto MM, Costa JR, Santos S. Freshwater decapod (*Aegla longirostri*) uses a mixed assessment strategy to resolve contests. Anim Behav. 2014 Sep 1;95:71–9.

60. Palaoro AV, Muniz DG, Santos S. Harder, better, faster, stronger: Weapon size is more sexually dimorphic than weapon biomechanical components in two freshwater anomuran species. J Morphol. 2020;281(9):1098–109.

61. Hendrickx ME, Hinojosa A, AyÃ³n-Parente M. In situ observations on the habitat and abundance of the squat lobster Gastroptychus perarmatus (Haig, 1968) (Crustacea: Decapoda: Chirostylidae) in the n orthern Gulf of California, Mexico. J Threat Taxa. 2013 Dec 26;5228–36.

62. Nizinski MS, McClain-Counts JP, Ross SW. Habitat utilization, demography, and behavioral observations of the squat lobster, Eumunida picta (Crustacea: Anomura: Eumunididae), on

western North Atlantic deep-water coral habitats. Deep Sea Res Part Oceanogr Res Pap. 2023 Mar 1;193:103953.

63. Wicksten MK. One to a customer: associations between squat lobsters (Chirostyloidea) and their anthozoan hosts. Mar Biodivers. 2020 Feb 4;50(1):14.

64. Ahyong ST, Roterman CN. Pristinaspinidae, a new family of Cretaceous kiwaiform stem-lineage squat lobster (Anomura, Chirostyloidea). Scr Geol. 2014;147(147):125–33.

65. Asakura A. The Evolution of Mating Systems in Decapod Crustaceans. In: Decapod Crustacean Phylogenetics. CRC Press; 2009.

66. Thiel M, Lovrich GA. Agonistic behaviour and reproductive biology of squat lobsters. In: The Biology of Squat Lobsters. CSIRO Publishing; 2011. p. 224–47.

67. Levin LA, Mendoza GF, Grupe BM, Gonzalez JP, Jellison B, Rouse G, et al. Biodiversity on the Rocks: Macrofauna Inhabiting Authigenic Carbonate at Costa Rica Methane Seeps. Sebastien Duperron, editor. Plos One. 2015 Jul 9;10(7):e0131080.

68. Parzefall J, Wilkens H. Zur ethologie augenreduzierter tiere. Untersuchungen an Munidopsis polymorpha Koelbel (Anomua Galatheidae). Ann Spéléologie. 1975;30:325–35.

69. Claverie T, Smith IP. Allometry and sexual dimorphism in the chela shape in the squat lobster Munida rugosa. Aquat Biol. 2010 Mar 9;8(2):179–87.

Supporting Information Captions

Table S1. Measurements (mm) for *Kiwa tyleri* body parts in this study to nearest 0.1 mm. CL= carapace length. PL = cheliped propodus length. PH = cheliped propodus height. CpL = cheliped carpus length. CpH = cheliped carpus height. M = male. F = female.

Table S2. Full linear model predictor coefficient output regarding the effect of log carapace length and sex (continuous and categorical predictor variables respectively) – or the interaction between the two – on log cheliped dimensions in the yeti crab species *Kiwa tyleri* and *Kiwa puravida*. Ten models were run. Initially models included interaction between log carapace length and sex, and if non-significant, a simpler model without the interaction term was run. Bonferroni corrections were performed, with significance below 0.005 designated with an asterisk. Std. Error = Residual standard error. DF = degrees of freedom. R^2 = regression model coefficient of determination. Adjusted R^2 = coefficient of determination adjusted for number of model predictors. F = F statistic. P1 = P-value indicating significance of linear model. B = the coefficient estimate for each predictor variable. Std. Error = the standard error of the estimate. t = the t-statistic, calculated as the estimate divided by the standard error. P2 = P-value indicating significance level of the predictor term in the model.

Figure 1

Click here to access/download;Figure;Figure_1a.tif

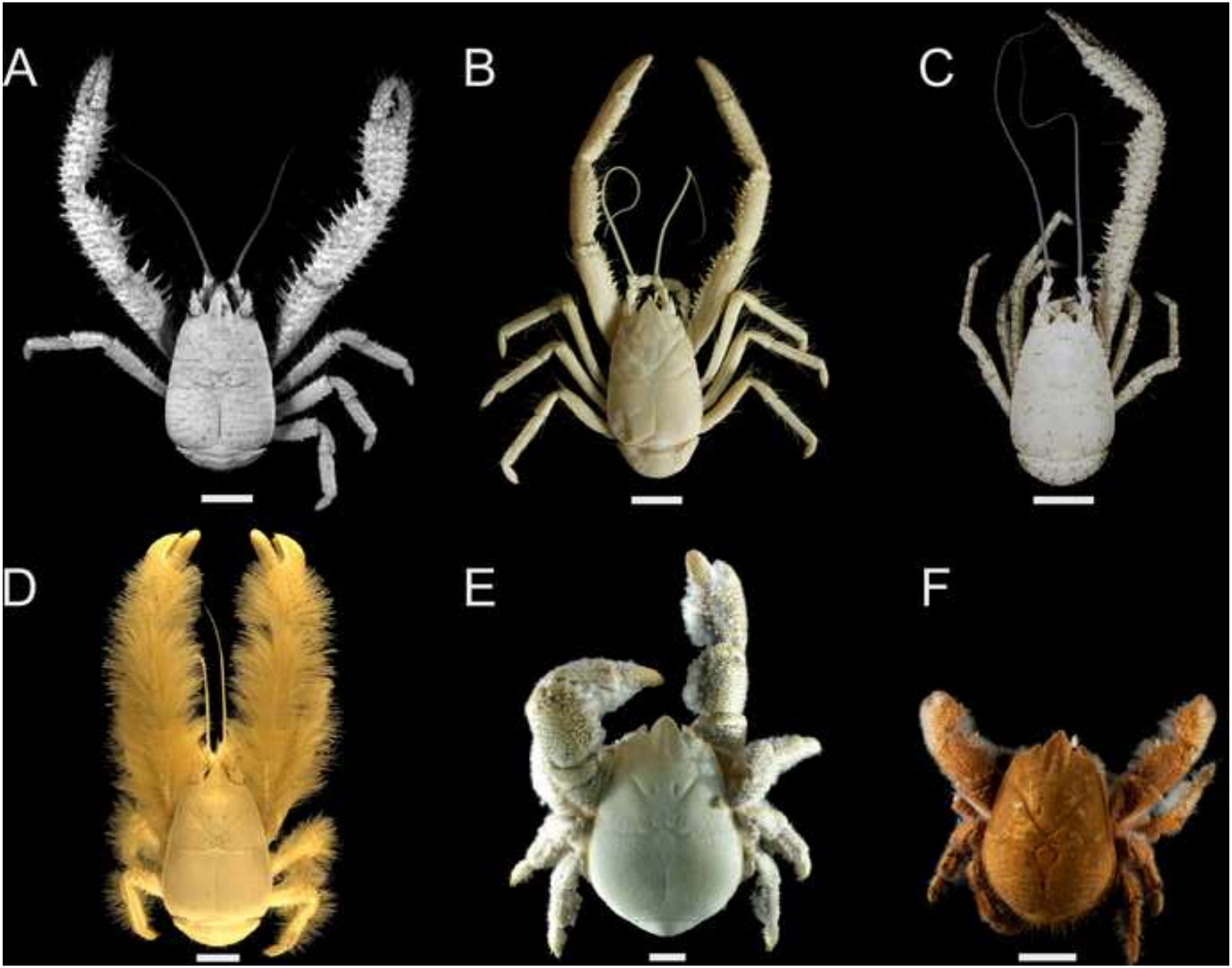

Figure 2

Click here to access/download;Figure;Figure_2.tif

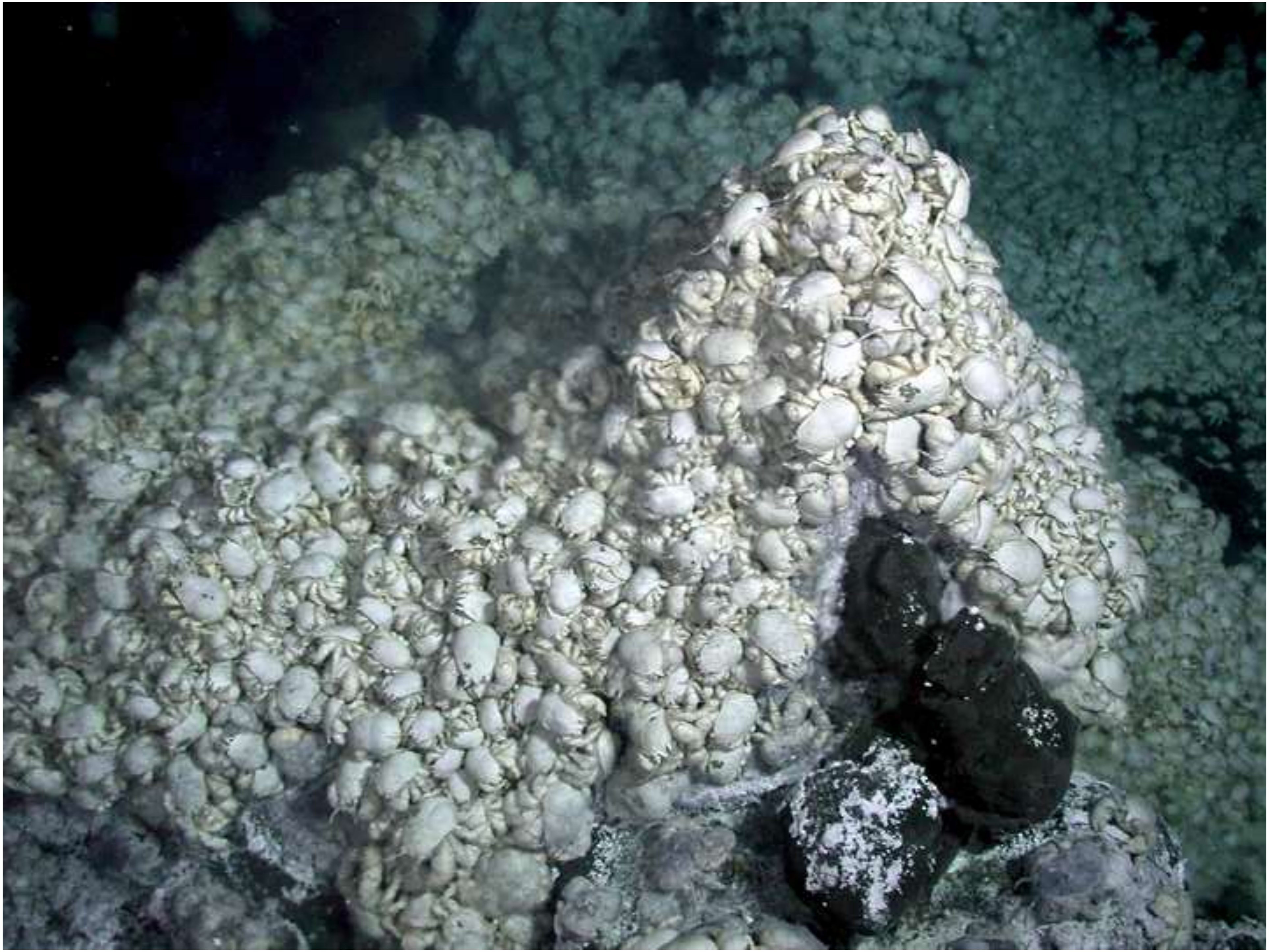

Figure 3

Click here to access/download;Figure;Figure_3.tif

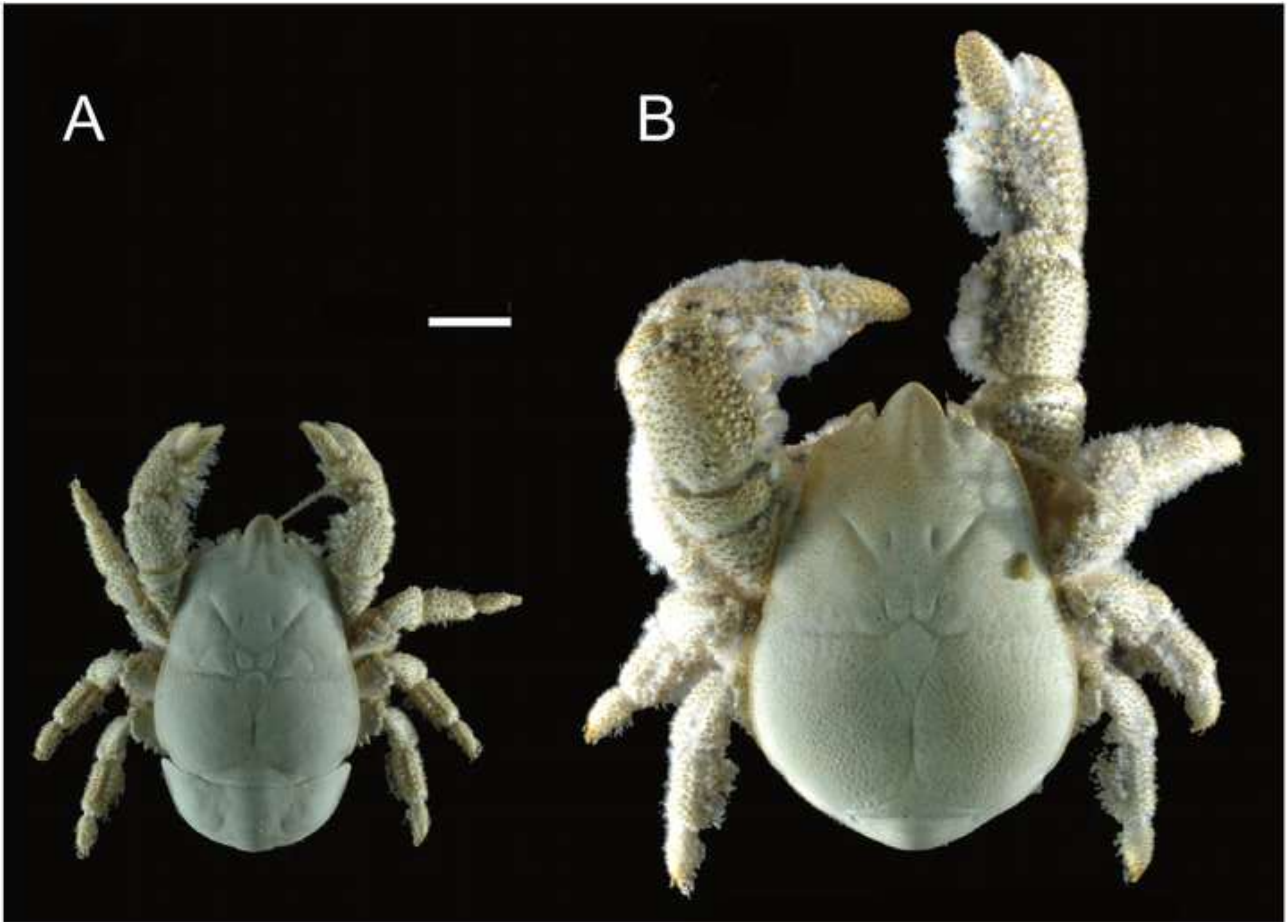

Figure 4

Click here to access/download;Figure;Figure_4.tif

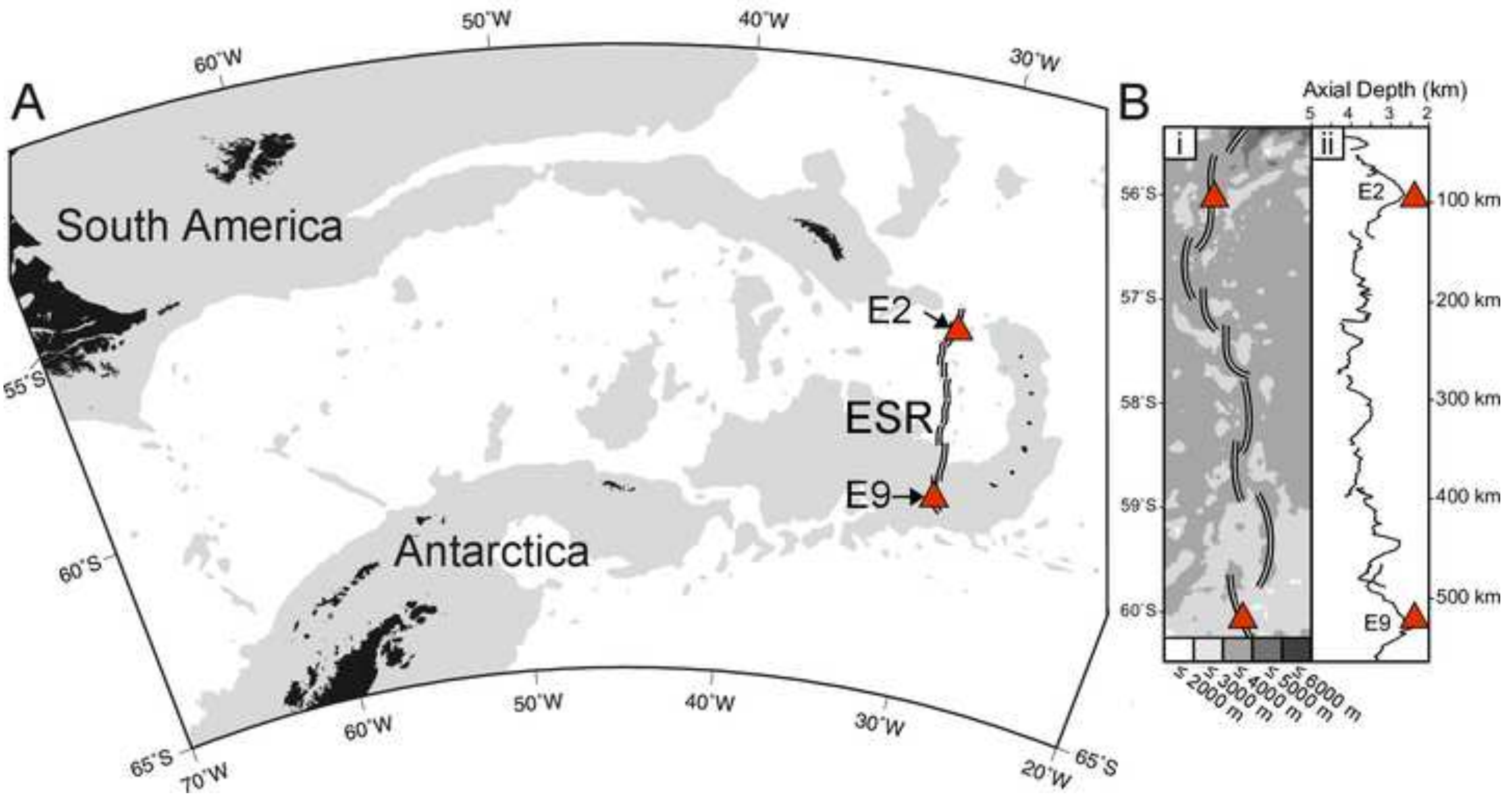

Figure 5

Click here to access/download;Figure;Figure_5.tif

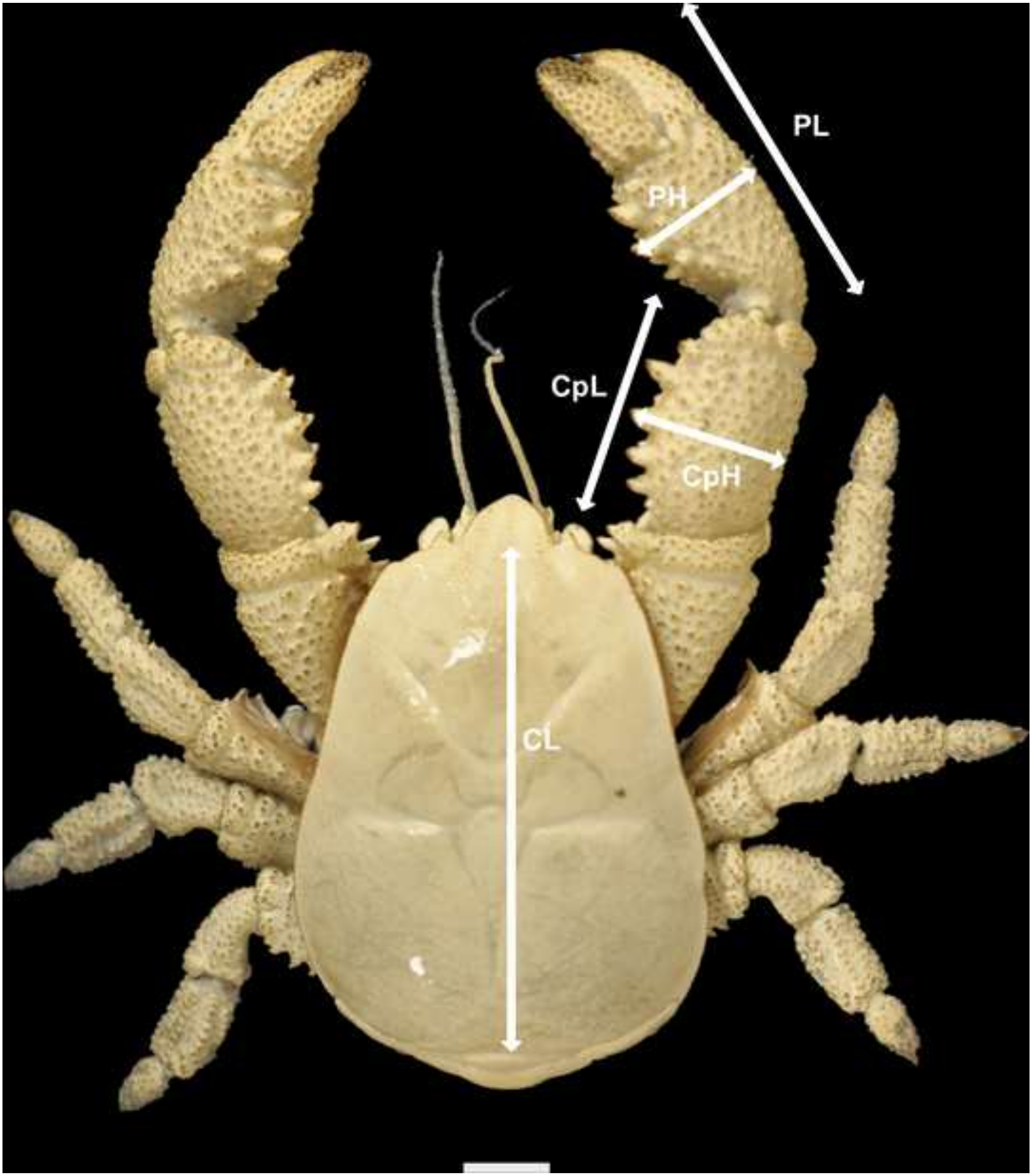

Figure 6

Click here to access/download;Figure;Figure_6.tif

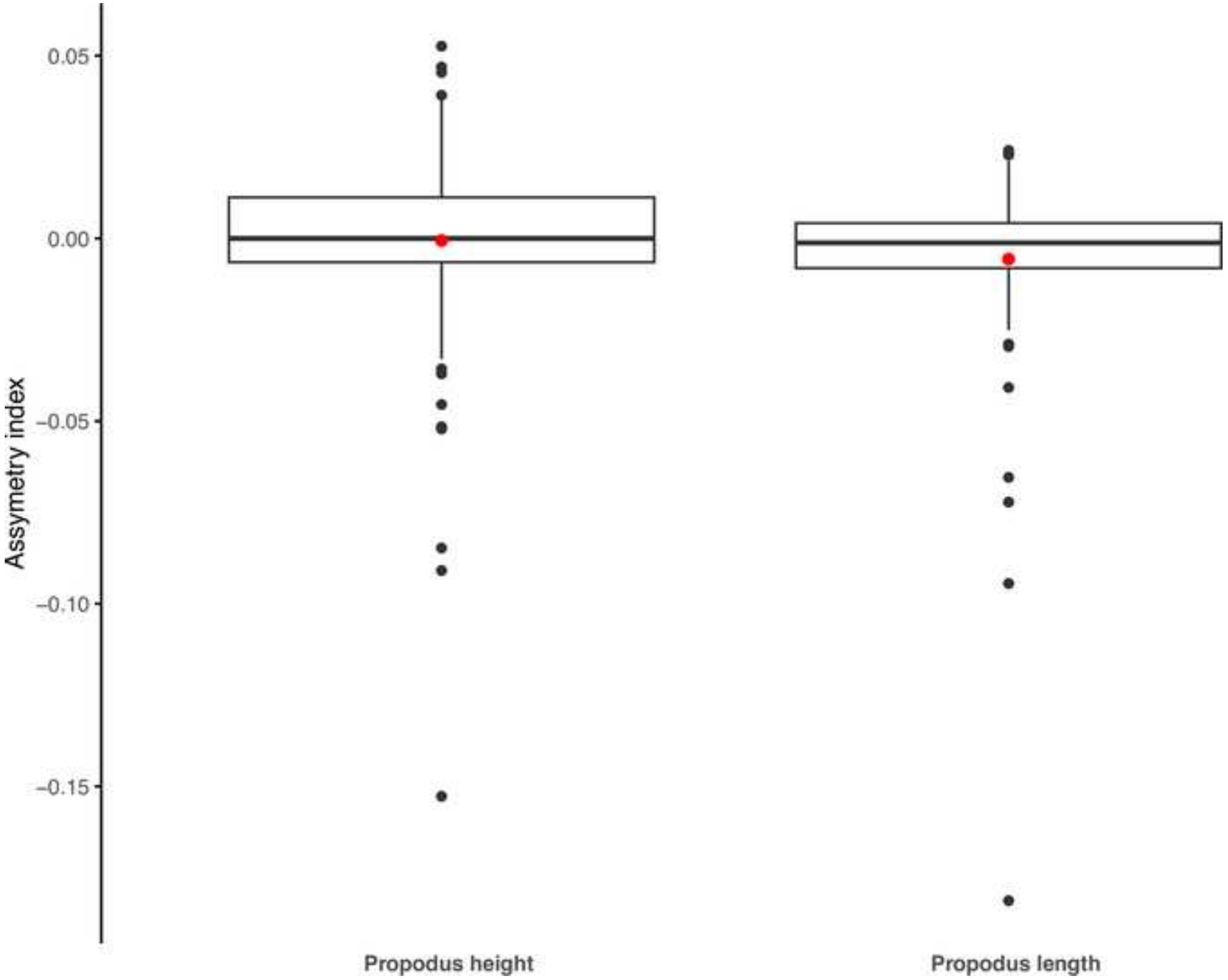

Figure 7

Click here to access/download;Figure;Figure_7.tif

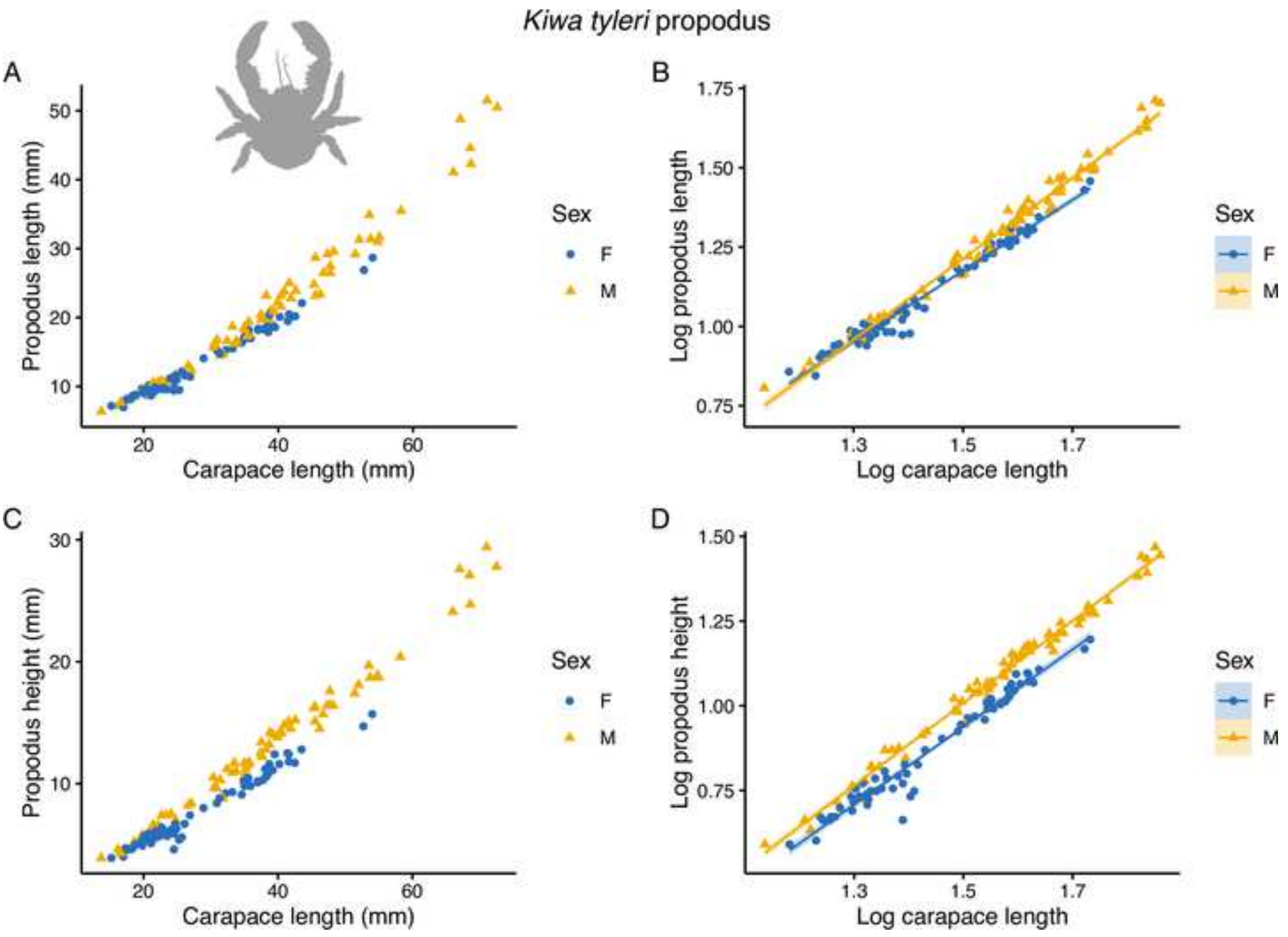

Figure 8 Click here to access/download;Figure;Figure_8.tif

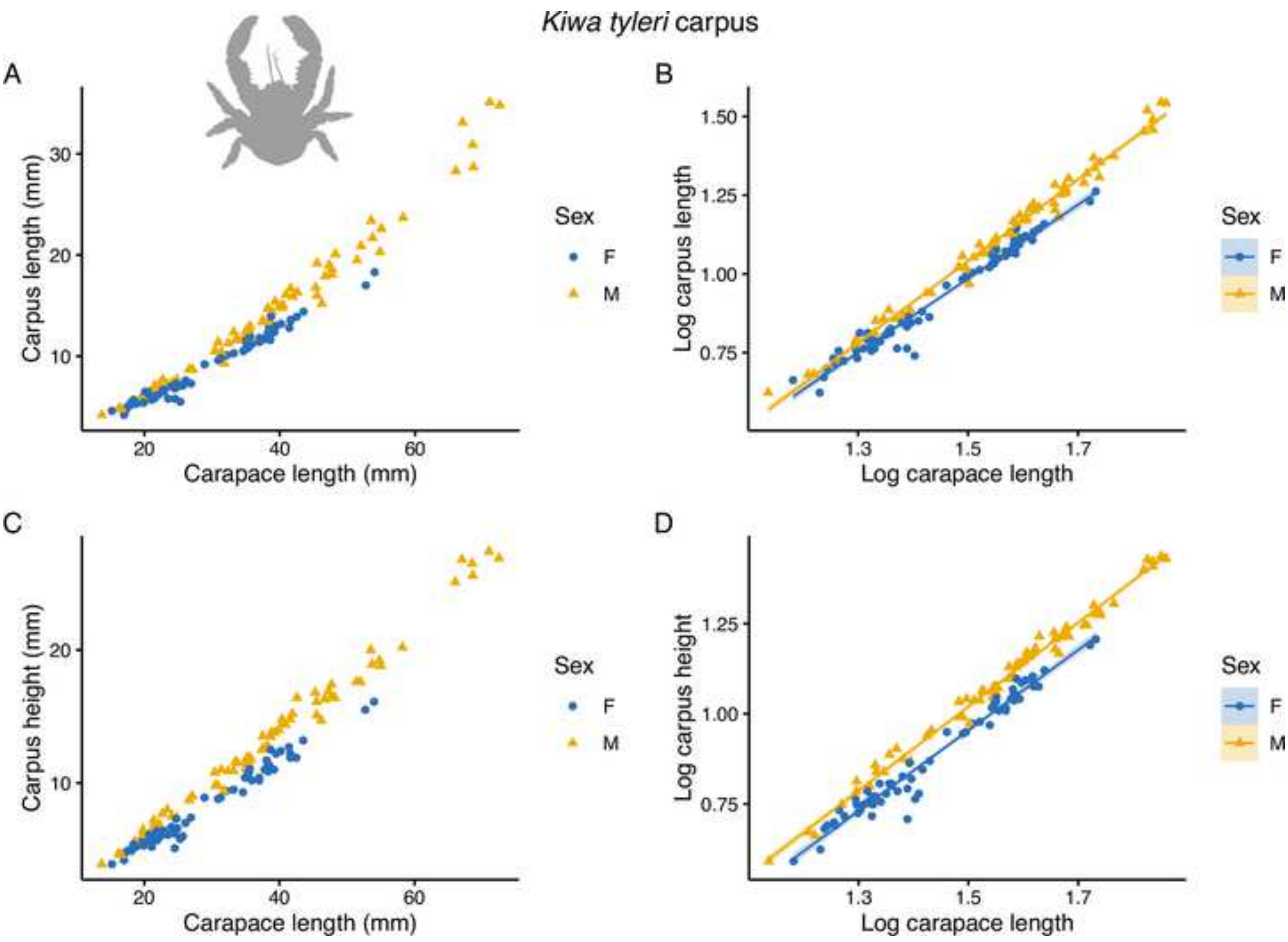

Figure 9 Click here to access/download;Figure;Figure_9.tif

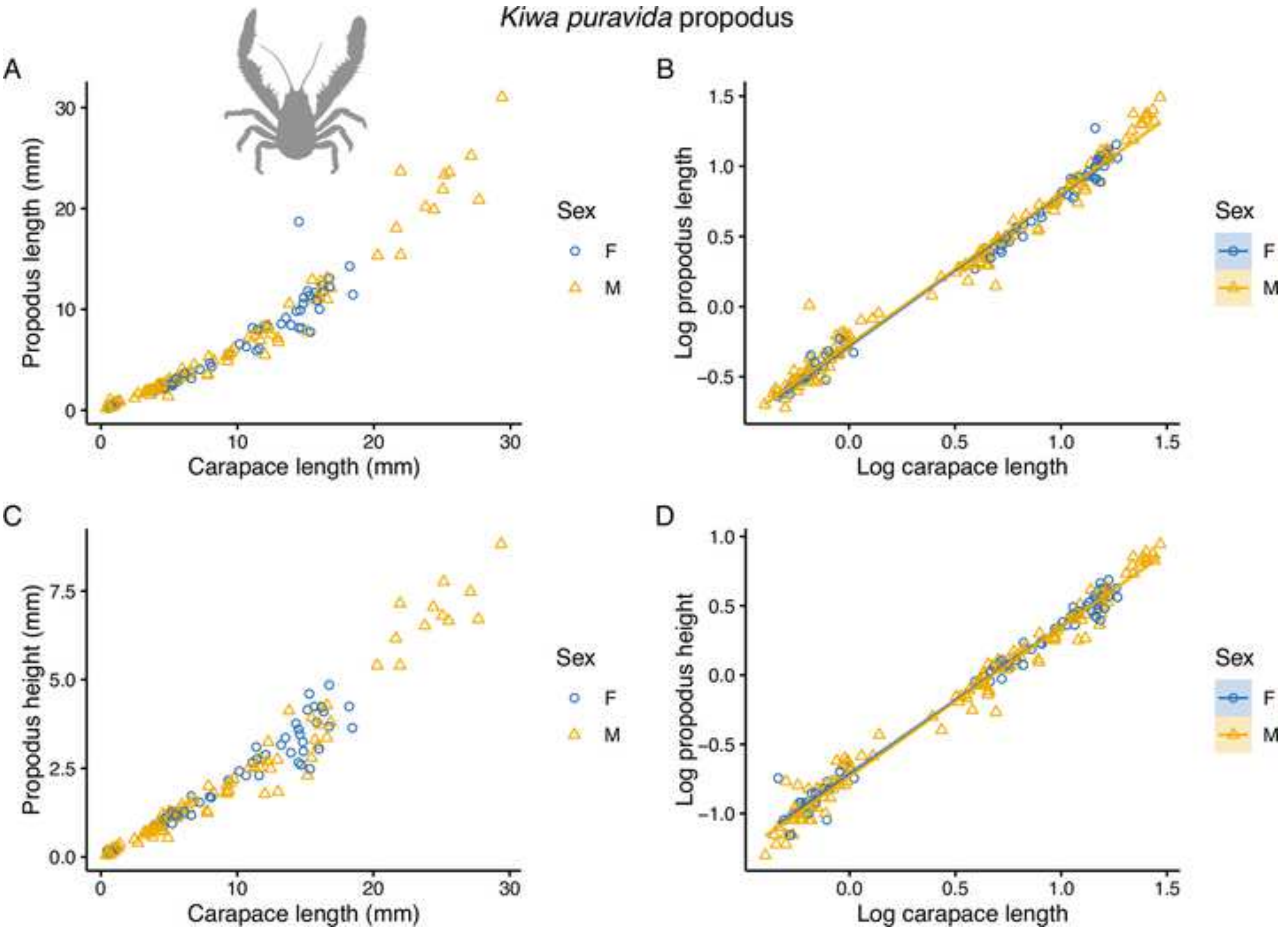

Click here to access/download
Supporting Information
Kiwa_dimorphism_supporting_information.docx
